# Toward an Ideal S = 1/2 Kagome Antiferromagnet and Quantum Spin Liquid Candidate through Selective Mg**^2+^** Doping of Averievite

**DOI:** 10.1021/acs.chemmater.6c01631

**Published:** 2026-09-24

**Authors:** Ethan Lowe, Lewis Giannelli, Claire Wilson, Ursula B. Hansen, David Boldrin

**Affiliations:** † School of Physics and Astronomy, 3526 University of Glasgow, University Avenue, Glasgow G12 8QQ, U.K.; ‡ Institut Laue Langevin, 71 Av. Des Martyrs, Grenoble 38000, France; § School of Chemistry, 3526 University of Glasgow, University Avenue, Glasgow G12 8QQ, U.K.

## Abstract

Quantum spin liquids (QSLs) are exotic states of matter where geometric frustration and quantum fluctuations suppress conventional magnetic order. S = 1/2 kagome antiferromagnets (KAFMs) are an ideal platform for realizing such behavior, and several mineral-inspired Cu^2+^-based kagome systems have emerged as promising candidates. Among these, Averievite (Cu_5_V_2_O_10_CsCl) offers a structurally distinct route to frustrated magnetism through its copper oxovanadate framework, which hosts alternating kagome and honeycomb layers. Prior studies have shown that targeted Zn^2+^ substitution on the honeycomb Cu_H_ site can suppress magnetic order and promote QSL-like behavior. Here we investigate Mg^2+^ substitution in the Averievite family, motivated by the geometric flexibility and favorable scattering contrast of Mg^2+^ relative to Zn^2+^, the previously explored diamagnetic dopant. Herein we present the novel Mg_*x*_Cu_5–*x*_V_2_O_10_CsCl (*x* = 0, 0.5, 1, 2) family, utilizing X-ray diffraction, single-crystal electron diffraction, and SQUID magnetometry to obtain precise structural parameters, including site occupancies, antisite disorder, and local coordination information, and to correlate these with the varied magnetic behavior. Increasing Mg^2+^ content leads to a pronounced suppression of both the structural and long-range magnetic ordering, alongside an increased mean field antiferromagnetic interaction, signaling enhanced magnetic frustration. Crucially, electron diffraction data is indicative of less than 1% Mg^2+^ antisite disorder onto the kagome lattice in the Mg_2_ compound. This contrasts with Zn-based analogues, preserving the integrity of the S = 1/2 kagome network. At *x* = 2, neither long-range order nor spin freezing is observed down to 2K, giving a new S = 1/2 KAFM with early hallmarks of QSL behavior and establishing Mg-doped Averievite as a clean and highly tunable platform for exploring QSL ground states.

## Graphical abstract

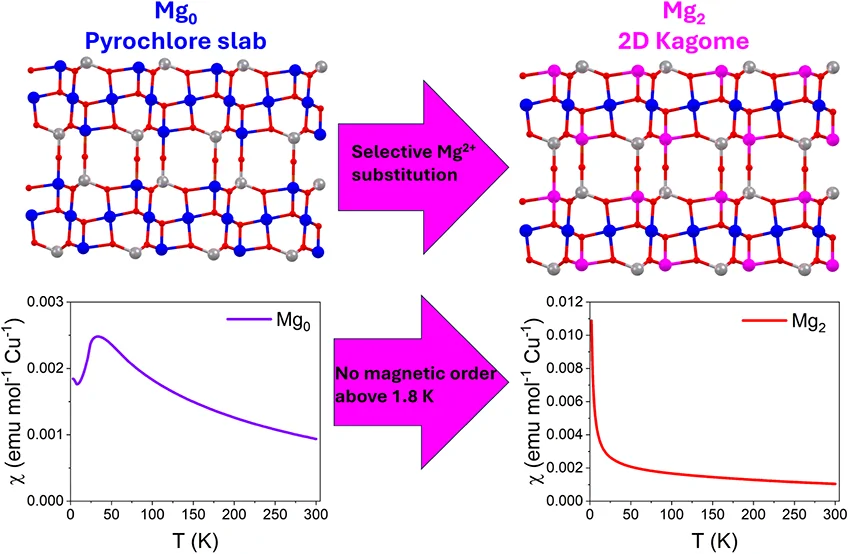

## Introduction

Quantum spin liquids (QSLs) are exotic states of matter where strong geometric frustration and quantum fluctuations result in a ground state that lacks conventional magnetic order.^1–3^ These states are of fundamental interest due to their ability to host long-range quantum entanglement^4^ and exotic quasi-particles such as spinons^5,6^ and give rise to unconventional superconductivity.^7^ S = 1/2 kagome antiferromagnets (KAFMs) provide a promising platform to realize QSL behavior due to their strong geometrically frustrated 2D exchange interactions and highly tunable coordination environments.^8^ While the unambiguous determination of a QSL state is still lacking, several exciting candidates in the S = 1/2 KAFM family inspired by naturally occurring minerals have been explored. Most studied are those closely related to the paratacamite and claringbullite families, including herbertsmithite,^9^ barlowite^10^ and their isostructural variants^11–19^ as well as other Cu^2+^ containing minerals, such as the pyrovanadate based vesignieite^20^ and volborthite.^21^ These materials have been the focus of sustained interest, as they have been shown to host a variety of flavors of QSL states beyond the archetypal resonating valence bond (RVB) states.^22–27^ As such, the generation of these *pseudo*-2D, spin-frustrated systems are highly promising candidates to investigate how subtle synthetic modification can generate interesting and potentially novel topological states.

Averievite (Cu_5_V_2_O_10_CsCl) is a recently discovered candidate to host an S = 1/2 KAFM, with its copper oxovanadate-based structure providing a notable difference to the hydroxide-based minerals previously discussed.^22,28^ It is a naturally occurring mineral that typically crystallizes in the trigonal space group *P*3̅*m*1 (no. 164) at ambient temperature. The structure can be visualized as either a CuO pyrochlore slab separated by CsO_2_ units or conversely as alternating kagome and honeycomb layers (Figure 1a–c). Upon cooling below ∼295 K, the structure undergoes a symmetry-lowering distortion to monoclinic *P*2_1_/*c* (no. 14), consistent with subtle lattice distortions that generate crystallographically inequivalent Cu^2+^ sites within the kagome plane.^22,29^ As such, inequivalent exchange occurs within the Cu^2+^ corner-sharing triangles, and the interplay between strong in-plane antiferromagnetic interactions within the kagome lattice and weaker interlayer exchange via the honeycomb layer gives rise to long-range antiferromagnetic order below T_N_ ≈ 24 K.

**Figure 1:**
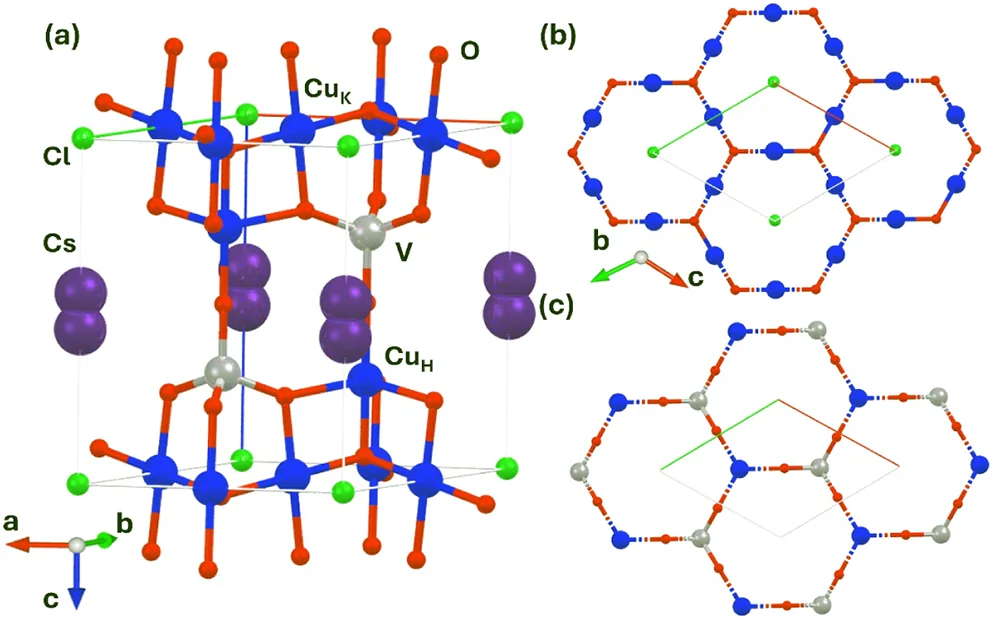
(a) The crystal structure of Averievite, highlighting its layered structure with (b) the Cl^–^-centered Cu_K_ Kagome plane and (c) the mixed Cu_H_/V honeycomb plane.

A number of similar synthetic analogues have been synthesized possessing modified structures through substitution of both metal and halide ions. A nonexhaustive list of these analogues is shown in Table 1, concentrating on magnetically characterized samples. While Averievite possesses magnetic ordering at ∼24 K, these analogues possess significant changes in both magnetic and structural phase transitions, highlighting the chemical flexibility of this family and a richness in their magnetic responses, making them an interesting family to explore frustration effects.^29–32^ While all analogues ultimately undergo a long-range magnetic ordering transition, with frustration indices of order 10, interestingly, the Weiss temperature varies considerably. Notably, the P-containing analogues show consistently lower magnitude values of ϑ, indicating that relatively subtle structural changes can drastically alter the magnitude or balance of superexchange.

**Table 1:** A Comparison of the Key Structural and Magnetic Parameters in the Undoped Averievite Family of Materials^a^

Sample	Cu_5_V_2_O_10_CsCl^22^	Cu_5_P_2_O_10_CsCl^29^	Cu_5_P_2_O_10_CsBr^29^	Cu_5_P_2_O_10_CsI^29^	Cu_5_V_2_O_10_CsBr^30^
Room temperature space group	*P*3̅*m*1	*P*3̅*m*1	*P*3̅*m*1	*P*3̅*m*1	*P*3̅
Structural transition (K)	295, 127^a^	12	75	230	130
T_N_ (K)	24	3.8	7	12.5	21
Θ (K)	–185	–48	–85	–119	–175
Frustration index	8	13	12	10	8

a A second structural transition which may be associated with incommensurate ordering within the CsCl channels.

Achieving an ideal S = 1/2 kagome lattice in Averievite requires targeted substitution of the honeycomb Cu_H_ site with a diamagnetic metal center. It has previously been shown that just 50% doping of the diamagnetic Zn^2+^ ion onto the honeycomb Cu_H_ site suppresses both the structural and magnetic phase transitions to at least 15 and 2 K, respectively, in Zn_1_Cu_4_V_2_O_10_CsCl (Zn_1_-Averievite).^22^ Further Zn^2+^ substitution results in the structure Zn_2_Cu_3_V_2_O_10_CsCl (Zn_2_-Averievite), which displays a complete suppression of structural and magnetic phase transitions to at least 2 K.^33^ Extensive characterization has been conducted on Zn_*x*_-Averievite compounds,^33–35^ and from inelastic neutron scattering (INS) experiments, Zn_2_-Averievite displays broad magnetic excitations that are indicative of a QSL state^33^ and show a similar energy dependence to the previously studied QSL candidate Herbertsmithite.^36,37^

While Zn_2_-Averievite is a promising S = 1/2 KAFM candidate, there are drawbacks associated with the use of Zn^2+^ as a diamagnetic ion, such as the antisite disorder of Zn^2+^ onto the kagome plane (ca. 7%). As such, we have decided to investigate doping of the Cu_H_^2+^ honeycomb site with another diamagnetic metal ion, Mg^2+^. Doping of Cu^2+^ sites with Mg^2+^ is not uncommon throughout solid-state synthesis due to their similar ionic radii and the relative abundance and availability of magnesium.^38–40^ However, the use of Mg^2+^ also offers the additional benefit of geometric flexibility compared to Zn^2+^ due to the purely ionic nature of the Mg–O bonding. As a result, Mg^2+^ is expected to adopt a trigonal bipyramidal geometry much more readily than Zn^2+^. Additionally, the large difference in X-ray and electron scattering length between Cu^2+^ and Mg^2+^ allows for a reliable determination of substitution levels using conventional laboratory analyses.

Herein we detail the synthesis, structural, and magnetic characterization of Mg_*x*_-Averievite (Mg_*x*_Cu_5–*x*_V_2_O_10_CsCl), where *x* = 0 (**Mg**_**0**_), 0.5 (**Mg**_**0.5**_), 1 (**Mg**_**1**_), and 2 (**Mg**_**2**_). Utilizing combined X-ray and microelectron diffraction, we obtain precise crystallographic information, such as site occupancies, antisite disorder, bond angles, and lengths, typically unobtainable from conventional laboratory PXRD data alone. This information, coupled alongside SQUID magnetometry, allows us to rationalize the changes in magnetic properties within this family of compounds through robust magneto-structural correlations. Crucially, we observe a significant suppression of both the structural and magnetic phase transitions present in the parent compound Averievite upon increased levels of Mg^2+^ doping, both of which are promising indicators of a highly frustrated state. Furthermore, the absence of any antisite disorder of Mg^2+^ onto the kagome lattice makes this family of compounds even more attractive S = 1/2 KAFM models.

## Synthesis

Mg_2_-Averievite (Mg_2_Cu_3_V_2_O_10_CsCl) was synthesized by adapting the previously reported syntheses.^20,22^ V_2_O_5_ (Sigma-Aldrich, 0.25 g, 1.37 mmol), CuO (Sigma-Aldrich, 0.3282 g, 4.122 mmol), MgO (Sigma-Aldrich, 0.1108 g, 2.74 mmol), and CsCl (Sigma-Aldrich, 0.243 g, 1.44 mmol) were ball milled in a planetary ball mill for 30 min at 250 rpm. The powder was split into four batches and pressed into four 10 mm pellets and heated in an alumina crucible at 600 °C for 48 h, cooled to 450 °C at 0.5 °C min^–1^, and then furnace cooled to room temperature. Mg_1_Cu_4_V_2_O_10_CsCl and Mg_0.5_Cu_4.5_V_2_O_10_CsCl were synthesized by replacing stoichiometric amounts of CuO with MgO (Alfa Aesar) at 4:1 and 4.5:0.5, respectively. For all materials, each synthesis produced ∼1 g of sample that was hand ground in a pestle and mortar for 10 min before further characterization.

## Results and Discussion

### Structural Analysis

Previous crystallographic studies of Averievite have shown it to crystallize in the trigonal *P*3̅*m*1 space group (no. 164) above 298 K. Upon cooling below 295 K, a symmetry-breaking transition to the monoclinic *P*2_1_/*c* space group (no. 14) is observed. This is followed by the onset of an unsolved but possible incommensurate phase below 127 K, likely associated with ordering of the Cs site.^22,33^ The structure is composed of vertex-sharing CuO_4_ square-planar units that assemble into two-dimensional kagome nets within the crystallographic *ab* plane (Figure 1). Within these layers, three crystallographically equivalent Cu^2+^ sites form a network of corner-sharing triangles, generating the characteristic kagome topology with short in-plane Cu_K_–O_K_–Cu_K_ superexchange pathways (∼115°).

The kagome layers are separated along the *c* axis by a mixed anionic honeycomb framework consisting of VO_4_ tetrahedra and interlayer Cu_H_O_5_ trigonal bipyramids. The latter provide a pathway for magnetic superexchange bridges between adjacent kagome planes, generating an interlayer coupling. Large Cs^+^ cations and Cl^–^ anions occupy two-dimensional channels that extend parallel to the *c* axis, stabilizing the layered architecture.

Initially we focus on room-temperature powder X-ray diffraction measurements. High-quality fits for all three doped compounds were obtained using the trigonal *P*3̅*m*1 space group, as observed in the parent structure Averievite,^22^ with structural and refinement parameters available in Table 2. Sample **Mg**_**0.5**_ displayed a small (∼3.85%) CuO impurity that was taken into account during the refinement. The refinement for **Mg**_**1**_ contained no impurity phases; however, a broadening of (h,0,3) peaks (*vide infra*) results in a slight reduction in the quality of fit (Figure S2). The refinement model for **Mg**_**2**_ includes two small CuO (1.47%) and Mg_3_V_2_O_8_ (1.78%) impurity phases and a <1% CsCl impurity, which was not washed out of the sample, as similar structures have reported a change in the magnetic properties upon washing.^29^ Full diffraction patterns and refinement results are presented in Figures S1–S3.

**Table 2:** Crystal Structure and Refinement Parameters Obtained at 298 K from Rietveld Refinement of X-ray Powder Diffraction Data for Mg*_x_* Cu_5–*x*_V_2_O_10_CsCl, Where *x* = 0.5, 1, 2

298 K XRD datasets	Mg_0.5_	Mg_1_	Mg_2_
System	Trigonal	Trigonal	Trigonal
Space group	*P*3̅*m*1	*P*3̅*m*1	*P*3̅*m*1
a (Å)	6.36261(11)	6.35643(18)	6.34281(10)
b (Å)	6.36261(11)	6.35643(18)	6.34281(10)
c (Å)	8.41103(10)	8.44180(19)	8.48382(8)
α (°)	90	90	90
β (°)	90	90	90
γ (°)	120	120	120
Volume (cm^–3^)	294.883(7)	295.387(13)	295.587(7)
Averievite phase %	96.15	100	96.04
Refined Mg_H_ %	15.8(7)	37.4(7)	78.4(4)
χ^2^	3.929	5.711	7.220
R_W_	4.244	5.396	4.868

To confirm that Mg^2+^ had been successfully doped onto the hexagonal Cu_H_ site, the fractional site occupancy was freely refined between Mg^2+^ and Cu^2+^ atoms, with equivalent positions and displacement parameters. Unlike with Zn^2+^ doping, this is possible to model using laboratory X-ray powder data due to the large differences in X-ray scattering factors between Mg^2+^ and Cu^2+^. Fractional occupancy of Mg^2+^ on the hexagonal Cu_H_ site was observed to increase with increased starting MgO amounts from 15.8 to 78.4% in **Mg**_**0.5**_ and **Mg**_**2**_, respectively (Table 2). Attempts to model antisite disorder of the Mg^2+^ ions onto the kagome plane by freely refining Mg and Cu occupancies resulted in an unstable refinement or reduced quality of fit. However, to assess whether the absence of Mg^2+^ substitution onto the Cu_K_ site can be justified by powder XRD data alone, we applied the Hamilton R-ratio test^41^ to **Mg**_**2**_, comparing a restrained model excluding Mg_K_ occupancy (R_wp_ = 4.868, *m* = 25) against an unrestrained model including one additional occupancy parameter (*m* = 26, *b* = 1). Mg_K_ was fixed at intervals across 1–50%, while all other parameters, including Mg_H_ occupancy, were refined freely, resulting in a shallow minimum at R_wp_ = 4.795 (Mg_K_ = 5%; Figure S4). With *n* = 7234, the critical ratio at α = 0.01 is 1.00046, and the observed ratio at the minimum R_wp_ = 1.0152 exceeds this threshold, indicating that inclusion of Mg on the Cu sublattice gives a statistically significant, if modest, improvement in fit.

However, this result should be interpreted with caution. Mg_H_ occupancy is strongly correlated with Mg_K_ occupancy, and across the scan, it increases monotonically to an unrealistic value of 1.107 (Figure S4), showing that the two parameters compensate for one another rather than being independently constrained by the data. The region identified as significant by the Hamilton R-ratio test (Mg_K_ ≈ 1–10%) coincides almost exactly with the range over which Mg_H_ can still compensate within physically allowed occupancy, rather than reflecting a reliable value for each site. We therefore do not regard this analysis as proof of Mg_K_ antisite disorder; it indicates that there is at most a small overall degree of Cu_H_/Mg_H_ site-mixing, which cannot be resolved from conventional powder XRD alone, highlighting the need for high-resolution single-crystal X-ray or neutron diffraction.

Clear differences in peak intensity and position are observed across the three analogues (Figures 2a, S5, and S6). A shift in the (1,0,1) and (1,1,0) peaks to higher 2θ highlights a contraction of the lattice parameters within the ab plane. A decrease in the relative intensity of the (1,1,0) plane with respect to the (1,0,1) plane is highly indicative of a reduced electron density on the hexagonal Cu_H_ site, further supporting the doping of Mg^2+^ only onto the Cu_H_ site (Figure S5). The observed reduction in the relative intensity of the (1,1,0) reflection with respect to the dominant (1,0,1) peak upon Mg^2+^ substitution is attributed to the lower atomic scattering factor of Mg^2+^ compared to Cu^2+^. As the atomic scattering factor is directly proportional to electron density, replacing the heavier Cu^2+^ (*Z* = 29) ions with lighter Mg^2+^ (*Z* = 12) ions decreases the local scattering. This selective attenuation of the (1,1,0) reflection occurs because the Cu_H_ sites contribute constructively to the structure factor (F_110_) of the (1,1,0) plane, making its relative intensity highly sensitive to the lower electron density of the dopant.

**Figure 2:**
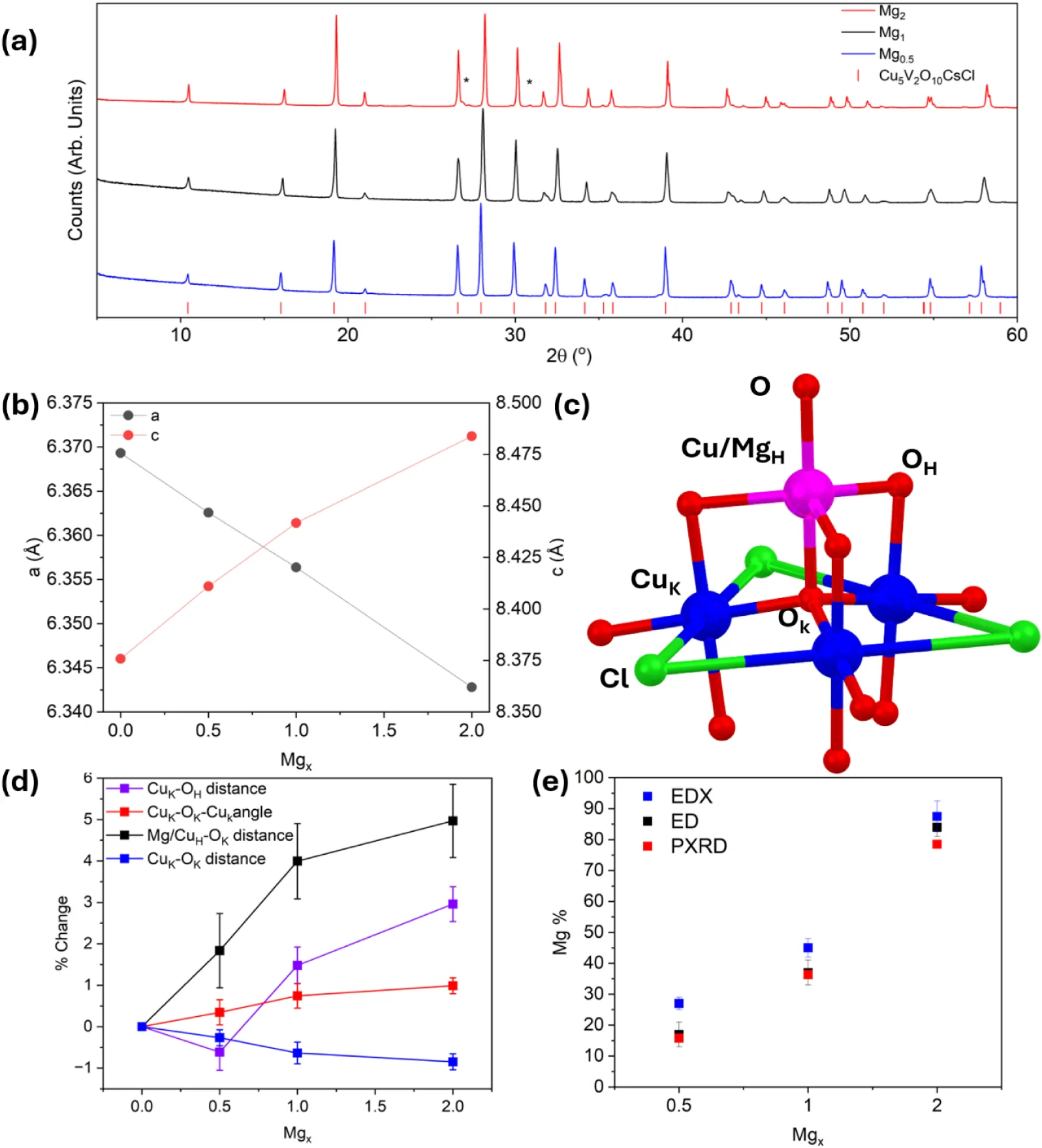
(a) Room temperature XRD patterns of **Mg_2_** (red), **Mg_1_** (purple), and **Mg_0.5_** (blue) from 5–100 2θ, where the two major Mg_3_V_2_O_8_ impurity peaks are denoted by *. (b) A comparison of the a, b, and c lattice parameters obtained from the PXRD refinements of **Mg_0.5_**, **Mg_1_**, and **Mg_2_**. (c) A fragment of the crystal structure of **Mg_0.5_** focusing on the Cu_4–*x*_Mg*_x_* O_11_Cl_3_ moiety. (d) A comparison of the % change in the ED determined bond lengths and angles across the Mg*_x_* family, with respect to the **Mg_0_**. (e) A comparison of the Mg % obtained from the PXRD refinements (black), electron diffraction (red), and EDX (blue) of **Mg_0.5_**, **Mg_1_**, and **Mg_2_**.

The above peak shifts are consistent with clear changes in lattice parameters and unit cell volume upon increased Mg^2+^ doping (Figures 2b and S6). A decrease in *a* and *b* lattice parameters is consistent with observations in the Zn_1_ and Zn_2_-averievite samples and can be rationalized by the slightly smaller size of the Mg^2+^ ion and may be indicative of stronger exchange interactions within a more tightly bound kagome plane. An increase in the *c* lattice parameter is also observed in Zn-doped analogues, which, in the case of Mg^2+^, can be attributed to increased Mg–O bond lengths arising from a purely electrostatic interaction (*vide infra*).

Interestingly, **Mg**_**1**_ displays a broadening of peaks corresponding to (h,0,3) lattice planes compared to **Mg**_**0.5**_ and **Mg**_**2**_ (Figure S6). This may be indicative of a nonhomogenous distribution of Mg^2+^ throughout the sample. However, further sintering of Mg_1_ results in no improvement in the broad peaks. While PXRD is indicative of this inhomogeneous distribution, direct probing of individual crystallites using electron diffraction will prove invaluable to analyze Mg^2+^ distribution throughout the sample.

Electron diffraction was employed to empirically determine the crystal structures of the synthesized compounds at the nanoscale while validating the model obtained from the bulk X-ray diffraction data. This technique permits reliable structure solution and provides access to single-crystal structural information from crystallites too small for conventional single-crystal X-ray diffraction. In addition, this technique is able to provide accurate information regarding bond angles and bond lengths that will prove invaluable in rationalizing changes in magnetism throughout this family of compounds. This capability is particularly significant within systems such as those for the present **Mg**_**1**_, where chemical doping is expected to induce small changes in electron density and displacement parameters that vary from crystal to crystal.

As such, we have analyzed a number of crystallites of **Mg**_**0.5**_, **Mg**_**1**_, and **Mg**_**2**_ at variable temperatures in order to obtain high-quality crystal structures, probe the effect of Mg^2+^ doping, and investigate the suppression of the trigonal to monoclinic phase transition. Additionally, we have obtained the first single-crystal electron diffraction structure of the pure Averievite structure, **Mg**_**0**_.

At 298 K, all four compounds are found to crystallize in the trigonal *P*3̅*m*1 space group (Table 3). For each sample, 4 crystals were examined and showed a consistent space group (*P*3̅*m*1) and unit cell parameters. The best datasets with 97.4, 98.3, 93.7, and 95.4% completeness and 0.80 Å resolution for **Mg**_**0**_, **Mg**_**0.5,**_ **Mg**_**1**_, and **Mg**_**2**_, respectively, were selected for structure solution and refinement with Olex2; see Table 3 and Figure S9, with full crystallographic data for each crystallite available in Tables S1–S8.

**Table 3:** Crystal Structure and Refinement Parameters Obtained at 300 K from Refinement of Single Crystal Electron Diffraction Data at 298 K for Mg*_x_* Cu_5–*x*_V_2_O_10_CsCl, Where *x* = 0, 0.5, 1, 2^a^

298 K ED datasets	Mg_0_	Mg_0.5_	Mg_1_	Mg_2_
System	Trigonal	Trigonal	Trigonal	Trigonal
Space group	*P*3̅*m*1	*P*3̅*m*1	*P*3̅*m*1	*P*3̅*m*1
a (Å)	6.3775(16)	6.3744(10)	6.3657(8)	6.3618(9)
b (Å)	6.3775(16)	6.3744(10)	6.3657(8)	6.3618(9)
c (Å)	8.3769(13)	8.3977(12)	8.4247(16)	8.4946(13)
α (°)	90	90	90	90
β (°)	90	90	90	90
γ (°)	120	120	120	120
Volume (cm^–3^)	295.07(13)	295.59(8)	295.65	297.74(8)
Refined Mg_H_ %	0	17(4)	35(4)^a^	84(3)
R1	12.04	14.49	12.23	12.92
wR2	30.93	34.67	29.66	30.01
Completeness	97.4	98.3	93.7	95.4
Identifier	2191	1806	1801	1781
CCDC number	2559045	2559047	2559051	2559053

a A distribution of Mg_H_% was obtained for different crystallites examined (*vide infra*).

As observed in the XRD data, a clear decrease in the *a* and *b* lattice parameters and an increase in *c* is observed upon increased Mg^2+^ doping (Table 3). The obtained Mg_H_ % for **Mg**_**0.5**_ and **Mg**_**2**_ samples indicates consistent levels of doping across multiple crystallites. Furthermore, attempts to refine any Mg^2+^ onto the Cu_k_ site resulted in unstable refinements where the Mg^2+^ site consistently possessed a negative occupancy. This is highly indicative of the absence of antisite disorder within the Mg_*x*_ family and a clear advantage of Mg^2+^ doping compared to the Zn_*x*_-Averievite family in the search for a QSL state. However, as reflections in a single-crystal dataset are much closer to statistically independent observations than points in a powder pattern, a Hamilton R-ratio test performed on single-crystal electron diffraction data provides a more rigorous check for significant Mg_K_ disorder. We therefore repeated the analysis using a single-crystal refinement of **Mg**_**2**_ (*n* = 253 reflections, *m* = 30), with a fixed Mg_K_ occupancy from 1 to 50% and Mg_H_ and all other parameters refined freely (Figure S10). As *n* – *m* is much smaller than in the powder case (223 vs 7208), the critical ratio is much larger, Rcrit = 1.0150 at α = 0.01. No dataset reaches statistical significance, indicating that no significant Mg_K_ occupancy is observed in **Mg**_**2**_. However, it should be noted that due to the use of a purely kinematic refinement model, this threshold is indicative rather than empirical.

The same correlation in Mg_H_ occupancy is observed in the data, with Mg_H_ increasing monotonically with fixed Mg_K_ to 0.934 at Mg_K_ = 50% (Figure S10). This consistency across two independent datasets and diffraction techniques supports the conclusion that the total level of Mg_K_ occupancy is small, although its exact magnitude from diffraction data alone is uncertain. Taken together, the powder X-ray and single-crystal electron diffraction data indicate, at most, a small degree of antisite Mg disorder into the kagome plane. A statistically significant improvement with up to 10% Mg_K_ occupancy in the powder X-ray diffraction data; however, this is highly correlated with the Mg_H_ occupancy. The single crystal analyses, despite the absence of a dynamical refinement, provide a further statistical test indicating that Mg incorporation ≥1% is not statistically significant.

As indicated by broad (h,0,3) peaks in the XRD pattern obtained for **Mg**_**1**_, a range of Mg content (0–35%) was found across the four crystallites examined, further confirming the inhomogeneous distribution of Mg^2+^ throughout individual crystallites (see Table S1). The reason for this variation is unclear; nonetheless, accessing this information via single-crystal ED provides crucial insights unobtainable from conventional characterization of solid-state samples and may prove an invaluable tool in the characterization of both the structural and electronic doping of QSL candidates.

Upon examination of the crystal structure of the Mg_*x*_ family, a number of key structural trends are observed, focusing primarily on the Cu_4–*x*_Mg_*x*_O_11_Cl_3_ fragment; see Figure 2c–d and Table 4. Importantly, we see a consistent increase in the Cu_K_–O_K_–Cu_K_ angle (115.23(30)–116.37(15)°) and a decrease in Cu_K_–O_K_ distance with increased Mg_H_%. According to Goodenough–Kanamori rules, we therefore expect an increase in the strength of the antiferromagnetic interactions between Cu_K_ sites. Accompanying these changes is an increase in the Cu_K_–O_H_ distance (1.959(7)–2.017(4) Å), which likely reduces the extent of the coupling between the kagome plane and hexagonal Mg/Cu_H_ site. An increase in the Mg/Cu_H_–O_K_ distance (1.852(13)–1.944(9) Å) can be attributed to the purely electrostatic interaction associated with Mg–O bonds and is expected to lead to a further decoupling of the magnetic interactions between the kagome planes.

**Table 4:** A Comparison of the Structural Parameters Obtained from the 298 K Single Crystal ED Refinement of Mg*_x_* Cu_5–*x*_V_2_O_10_CsCl, Where *x* = 0, 0.5, 1, 2^a^

	Mg_0_	Mg_0.5_	Mg_1_ ^a^	Mg_2_
Cu_K_–O–Cu_K_ angle (deg)	115.23(30)	115.63(18)	116.09(17)	116.37(15)
Cu_K_–O_K_ distance (Å)	1.888(3)	1.883(2)	1.876(2)	1.872(2)
Mg/Cu_H_–O_K_ distance (Å)	1.852(13)	1.886(10)	1.926(12)	1.944(9)
Cu_K_–O_H_ distance (Å)	1.959(7)	1.947(5)	1.988(6)	2.017(4)

a Values have been averaged across different crystallites measured.

To assess the effect of Mg^2+^ doping on the *P*3̅*m*1 to *P*2_1_/*c* structural transition, variable temperature ED measurements were carried out on **Mg**_**0**_, **Mg**_**0.5**_, and **Mg**_**2**_. Variable temperature measurements were excluded for **Mg**_**1**_ due to the variation in Mg^2+^ doping throughout different crystallites. As expected, the well-studied **Mg**_**0**_ analogue displays full conversion to the monoclinic *P*2_1_/*c* space group at 250 K (Tables S2 and S6). **Mg**_**0.5**_ possesses a consistent space group (*P*3̅*m*1) and unit cell parameters at both 298 and 250 K, confirming a suppression of the structural transition despite only low levels of doping (Tables S3 and S6). **Mg**_**2**_ displays a consistent space group (*P*3̅*m*1), unit cell parameters, and site occupancies across all measured temperatures: 300, 200, and 100 K (Table S4). The absence of a structural transition at the lowest temperatures available to this ED setup highlights the viability of Mg^2+^ doping as a novel strategy to suppress structural transitions within the Averievite family and thus retain the high symmetry trigonal structure at lower temperatures.

Scanning electron microscopy-energy dispersive X-ray (SEM-EDX) analysis was conducted as a complementary technique to corroborate Mg^2+^ doping, probe elemental distribution, and crystallite size and morphology. For each sample, three different sites were probed to ensure homogeneity of the sample (Figure S11). Due to the inherent insensitivity of EDX to oxygen, the mass % was fixed to the value calculated from the single-crystal ED structures obtained (21.39%). The atomic percentages of each element were highly consistent across the 3 sites examined for each sample, with an average atomic % across the 3 sites shown in Table S9. We suggest that the consistent atomic percentages found in **Mg**_**1**_ are due to the limited resolution available from EDX, further highlighting the importance of single-crystal electron diffraction measurements. Taking the ratio of Cu^2+^:Mg^2+^, we obtain structural formulas of Mg_0.54_Cu_4.46_V_2_O_10_CsCl, Mg_0.90_Cu_4.10_V_2_O_10_CsCl, and Mg_1.75_Cu_3.25_V_2_O_10_CsCl for **Mg**_**0.5**_, **Mg**_**1**_, and **Mg**_**2**_, respectively.

A comparison of the Mg_H_^2+^ % across the three separate techniques, EDX, ED, and PXRD, is shown in Figure 2e. Taking an average of the three techniques results in approximate Mg fractions of 19.9, 39.5, and 84.3% for **Mg**_**0.5**_, **Mg**_**1**_, and **Mg**_**2**_, respectively. A combination of these techniques clearly shows an increased Mg % with increased MgO doping; furthermore, ED and XRD refinements are highly indicative of Mg^2+^ doping exclusively on the Cu_H_ site rather than in the kagome plane. As such, we have used average Mg^2+^ occupancy values across the three techniques for further analysis using SQUID magnetometry, with the expected molecular formulas Mg_0.40_Cu_4.60_V_2_O_10_CsCl, Mg_0.79_Cu_4.21_V_2_O_10_CsCl, and Mg_1.69_Cu_3.31_V_2_O_10_CsCl for **Mg**_**0.5**_, **Mg**_**1**_, and **Mg**_**2**_, respectively.

### SQUID Magnetometry

To probe the evolution of the antiferromagnetic transition with Mg%, SQUID magnetometry was employed to study the temperature and magnetic field dependence in the Mg_*x*_ family. Zero-field-cooled and field-cooled magnetic susceptibility measurements were conducted on samples **Mg**_**0**_–**Mg**_**2**_ under a 500 Oe magnetic field and across a 2–300 K temperature range. Measurements reveal a clear evolution of magnetic behavior across the four samples (Figures 3a and S12).

**Figure 3:**
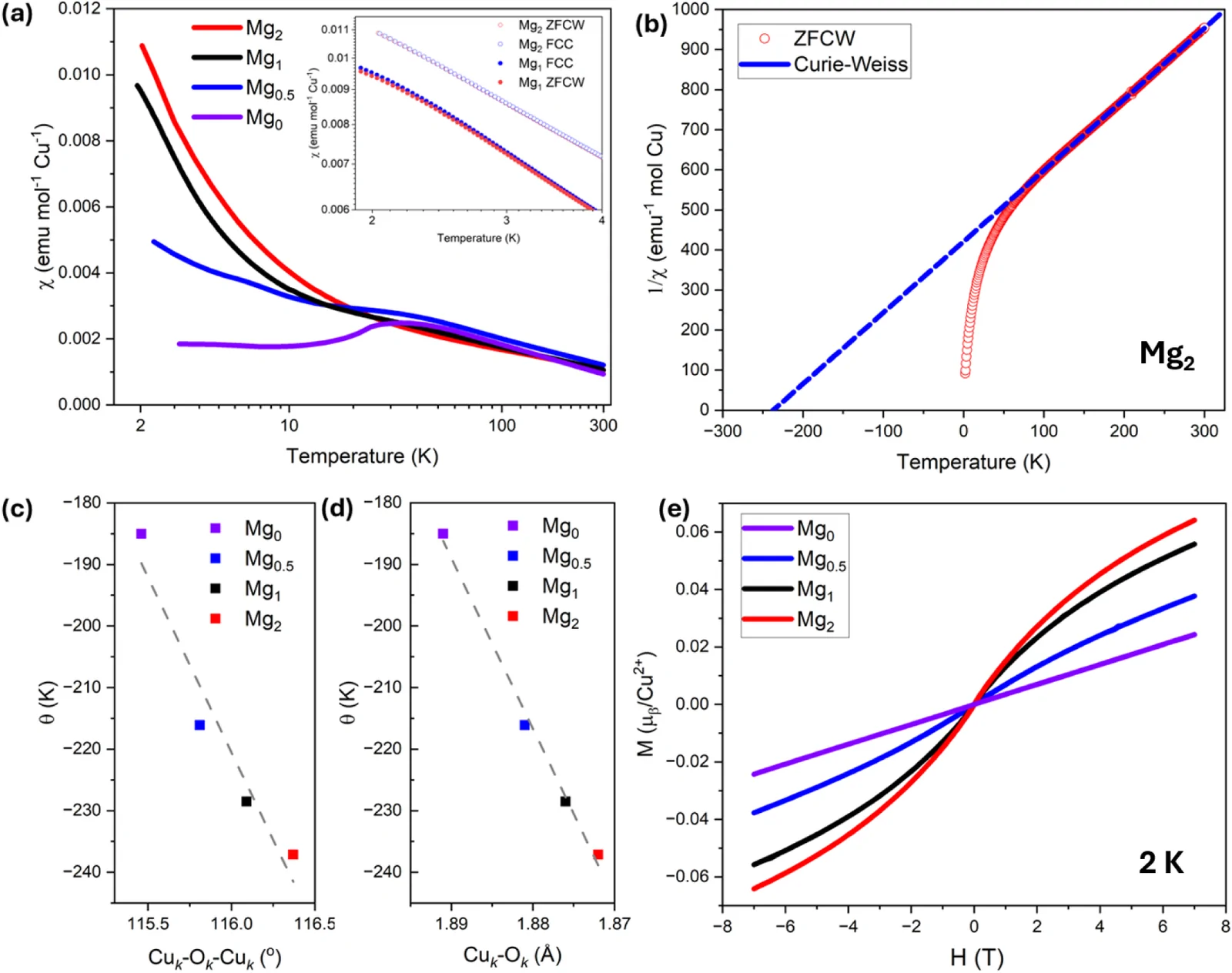
(a) The field-cooled magnetic susceptibility of Mg_0_–Mg_2_ and field-cooled vs zero-field-cooled magnetic susceptibility for Mg_1_ and Mg_2_ zoomed-in from 2 to 4 K (inset). (b) The Curie–Weiss plot of 1/χ vs T for Mg_2_. (c) A comparison of changes in Cu_k_–O_k_–Cu_k_ bond angle vs Weiss constant for different Mg*_x_* samples; gray dashed line is a guide for the eye. (d) A comparison of changes in Cu_k_–O_k_ bond length vs Weiss constant for different Mg*_x_* samples. (e) Magnetization vs field plots at 2 K for compounds Mg_0_, Mg_0.5_, Mg_1_, and Mg_2_.

The parent compound Mg_0_,^22^ χ exhibits a pronounced maxima at T ≈ 25 K, indicative of a transition to long-range antiferromagnetic order. Above the transition, the inverse susceptibility follows Curie–Weiss behavior, yielding a negative Weiss constant (−185 K) consistent with prevailing antiferromagnetic interactions. In contrast, progressive chemical doping in **Mg**_**0.5**_, **Mg**_**1**_, and **Mg**_**2**_ systematically suppresses this antiferromagnetic transition (Figure 3a). This is highlighted by only a slight kink in the χ vs T in **Mg**_**0.5**_ and the complete disappearance of the peak in χ vs T in **Mg**_**1**_ and **Mg**_**2**_. This is consistent with the substitution of S = 1/2 Cu^2+^ centers from the hexagonal Cu_H_ site for diamagnetic Mg^2+^ ions, determined by diffraction, resulting in more isolated Cu_k_ kagome planes and an absence of strong interlayer coupling. Curie–Weiss fits to the inverse susceptibility further indicate an enhanced frustration due to a decreasing Weiss constant (Θ) with Mg_H_%, from −216.1(3) K to −237.1(3) K for **Mg**_**0.5**_ to **Mg**_**2**_; see Figure 3b–d, S13 and Table 5. As proposed in the structural section, this trend is consistent with the increased Cu_K_–O_K_–Cu_K_ angles within the kagome planes of the Mg_*x*_ doped analogues (Figure 2d). This increased Cu_K_–O_K_–Cu_K_ angle, away from 90°, generates an enhanced contribution to antiferromagnetic exchange. Interestingly, as a function of Zn-doping, the opposite trend in theta was observed for Zn_*x*_-Averievite samples, despite larger Cu_K_–O_K_–Cu_K_ angles and shorter Cu_K_–O_K_ distances observed in the Zn_2_ analogue.^33^ Given that in both cases we would expect enhanced Cu_k_–Cu_k_ AFM exchange, the opposite trends of theta suggest subtle differences in how further neighbor exchanges (e.g., interlayer) are modified by Mg^2+^ and Zn^2+^ doping.

**Table 5:** A Comparison of the Magnetic Properties of **Mg_0_**, **Mg_0.5_**, **Mg_1_**, and **Mg_2_**, Obtained from SQUID Magnetometry

	Mg_0_	Mg_0.5_	Mg_1_	Mg_2_
C (emu mol^–1^ K Cu^–1^)	0.525 ^22^	0.614(4)	0.560(1)	0.563(3)
Θ (K)	–185 ^22^	–216.1(3)	–228.5(1)	–237.1(3)
μ_eff_ (μ_β_/Cu^2+^)	2.05 ^22^	2.22	2.12	2.12
χT (emu mol^–1^ K Cu^–1^)	–	0.357	0.318	0.314
μ_eff_ (μ_β_) (300 K)	–	1.69	1.59	1.59

The μ_eff_ values obtained from a fitting of the Curie–Weiss data fall within 2.12–2.22 μ_β_ (Table 5) and are characteristic of a single S = 1/2 Cu^2+^ spin, correlating with other Averievite systems in the literature.^22,29,30^ The larger than expected μ_eff_ values obtained from the Curie–Weiss fits are extremely common in other kagome systems (1.78(2)–2.39(1) μ_β_) and are likely due to the combined presence of spin–orbit coupling and excited and ground state mixing under a magnetic field.^22,29,30^ Further analysis of the χ_T_ vs T plots (Figure S14) reveals room temperature χ_T_ values of 0.362, 0.318, and 0.314 cm^3^ mol^–1^ K Cu^–1^ for **Mg**_**0.5**_, **Mg**_**1**_, and **Mg**_**2**_, respectively. The room temperature χ_T_ values are around the expected value of 0.375 cm^3^ mol^–1^ K Cu^–1^ for a single uncoupled Cu^2+^ ion. The slight reduction from the expected may be attributed to ligand field effects associated with the Cu^2+^ centers.^42^

Magnetization vs field data were collected for **Mg**_**0.5**_, **Mg**_**1**_, and **Mg**_**2**_ at 2 K up to a magnetic field of 7 T (Figure 3e). A clear decrease in the linearity of the response, expected for an antiferromagnet, is observed moving from **Mg**_**0**_ to **Mg**_**2**_. This further confirms the suppression of the antiferromagnetic transition. The response of **Mg**_**2**_ is similar to that observed in other S = 1/2 KAFM systems, e.g., Herbertsmithite and Zn_2_-Averievite (**Zn**_**2**_), where two distinct contributions to the magnetization are present, the intrinsic kagome contribution and a paramagnetic component arising from remaining impurity Cu^2+^ ions. Fitting the 2K M­(H) data in Figure 3e to extract the balance of these two components gives a lower paramagnetic component in **Mg**_**2**_ compared to **Zn**_**2**_ (see SI Figure S16 and the SI for the fit details). Consequently, this suggests a slightly decreased number of defect spins in **Mg**_**2**_ based on this analysis; however, we note that higher field measurements would provide a more accurate deconvolution of these two contributions.

At low temperatures, **Mg**_**1**_ displays a slight divergence of ZFC and FC susceptibilities, which may be indicative of a low-temperature glassy phase, as observed in Zn_1_-Averievite, Figure 3a (inset).^22^ Importantly, the absence of a bifurcation in the field-cooled and zero-field-cooled susceptibility of **Mg**_**2**_ down to 2 K highlights a potential absence of this glassy phase in addition to the suppression of long-range magnetic order. This is a hallmark of spin-frustrated QSL candidates and suggests the viability of **Mg**_**2**_ for further experimental probes for QSL indicators through techniques such as INS and μ_SR_. Collectively, these results further demonstrate the rich chemical flexibility of the Averievite family, which provides multiple routes to enhance frustration with subtle differences in the bulk magnetic properties.

## Conclusions

In this work, we report the successful synthesis and in-depth characterization of a new family of S = 1/2 kagome antiferromagnets achieved through selective Mg^2+^ doping on the honeycomb sublattice of Averievite. This chemical strategy provides a viable route to tuning magnetic interactions in geometrically frustrated systems and offers a new platform for the exploration of QSL behavior. A key advance of this study lies in the structural determination of individual crystallites using single-crystal electron diffraction (ED). The use of ED enables precise structural determination, which is inaccessible or highly challenging to resolve via conventional powder X-ray diffraction,^29^ providing critical information for rationalizing the evolution of magnetic interactions within the series. We find that increasing Mg^2+^ substitution systematically enhances mean field antiferromagnetic interactions, consistent with subtle but significant modifications to the local bonding environment of both the kagome and honeycomb layers. This strengthening of antiferromagnetic coupling is accompanied by a pronounced suppression of both magnetic and structural phase transitions in the Mg_*x*_ family, indicating the progressive destabilization of long-range order in favor of a disordered ground state. Importantly, our structural analysis indicates minimal statistically significant antisite disorder of Mg^2+^ within the kagome planes above 1% for the single-crystal electron diffraction datasets; however, future dynamical refinements and single-crystal neutron studies are required to strengthen this interpretation. The absence of disorder or defects within the kagome plane is essential for the realization of a true QSL state. These results identify the Mg_*x*_-Averievite family as highly promising candidates in the ongoing experimental search for QSL systems. Future work on these materials will focus on probing the low-temperature dynamic properties and excitations using advanced spectroscopic and scattering techniques, with the aim of further characterizing the Mg_*x*_ family as viable QSL candidates.

## Methods

Powder X-ray diffraction (PXRD) data were collected on freshly prepared samples of **Mg**_**0.5**_, **Mg**_**1**_, and **Mg**_**2**_ using a Malvern Panalytical Empyrean multipurpose diffractometer (University of Glasgow) equipped with a sealed-tube Cu Kα radiation source (λ = 1.5406 Å) operating at room temperature. Samples were prepared as dark-brown microcrystalline powders and mounted on zero-background silicon sample holders. Diffraction patterns were recorded over the range of 5–100° 2θ using a step size of 0.131° and a scan rate of 0.2639° min^–1^. Phase analysis and quantitative refinement were performed using GSAS-II fitted to a Chebychev-1 background with six background coefficients. Hamilton R-ratio tests used to assess site occupancy models are described in the main text and Supporting Information.

For three-dimensional electron diffraction (3D ED), dry powders were deposited onto 200-mesh copper transmission electron microscopy grids coated with lacey ultrathin amorphous carbon and gently tapped to remove excess powder from the grid. Data were collected from individual crystallites using a Rigaku XtaLAB Synergy-EDS1 equipped with a LaB_6_ electron source operating at 200 kV (λ = 0.0251 Å) under high vacuum (∼10^–5^ Pa) using a Gatan Elsa cryoholder. Measurements were performed at 100, 200, 250, and 300 K following cooling of the mounted samples within the microscope. Continuous-rotation datasets covering approximately 120° were collected from at least four crystallites per sample at each temperature using a Rigaku HyPix-ED detector, with a rotation increment of 0.25° per frame and an exposure corresponding to 2 s deg^–1^, using a selected-area aperture with an apparent diameter of 2 μm. Diffraction data were indexed, integrated, and reduced using CrysAlisPro for ED. Structures were refined by least-squares methods using SHELXL and Olex2.refine through Olex2 under the kinematical approximation with electron scattering factors. Extinction parameters, anisotropic displacement parameters, and Mg/Cu occupancies were refined as described in the Supporting Information. Crystallographic details are provided in the deposited CIF files. CCDC deposition numbers 2559045–2559054 and 2558898 contain the supplementary crystallographic data for this work.

Variable-temperature DC and AC magnetic measurements were performed using a Quantum Design MPMS3 SQUID magnetometer (University of Glasgow). Finely ground microcrystalline samples were loaded into gelatin capsules supported in plastic straws. Diamagnetic contributions from the sample holder were determined from blank measurements, and core diamagnetism was corrected using Pascal’s constants. DC magnetic susceptibility data were collected over 2–300 K in an applied field of 500 Oe under zero-field-cooled and field-cooled conditions. Isothermal magnetization measurements were collected over the field range −7 to 7 T at 2 K. Molar magnetic susceptibilities were calculated from the measured magnetic moments using the molecular formula weights determined from the refined crystal structures.

## Supplementary Material



## Notes

Supplementary structural characterization, including Rietveld refinement results, Hamilton R-ratio test details, additional electron diffraction data and images, crystallographic tables, EDX data and images, and SQUID magnetometry data (PDF)
