# Supplementary material for: Toward an Ideal S = 1/2 Kagome Antiferromagnet and Quantum Spin Liquid Candidate through Selective Mg 2+ Doping of Averievite
Source: Chem Mater. 2026 Sep 24;38(19):10085–94. doi: 10.1021/acs.chemmater.6c01631 (PMC13673998; doi:10.1021/acs.chemmater.6c01631)
Supplement: Supplementary file 1 [file cm6c01631_si_001.pdf]

Towards an ideal S = ½ kagome antiferromagnet and quantum spin liquid candidate through selective Mg^{2+} doping of averievite

Ethan Lowe* [1], Lewis Giannelli [1,2], Claire Wilson [3], Ursula B. Hansen[2], David Boldrin [1]

1. School of Physics and Astronomy, University of Glasgow, University Avenue, Glasgow G12 8QQ, U.K. E-mail: ethan.lowe@glasgow.ac.uk

2. Institut Laue Langevin, 71 Av. des Martyrs, 38000, Grenoble, France.

3. School of Chemistry, University of Glasgow, University Avenue, Glasgow G12 8QQ, U.K.

Experimental Methods

Powder X-ray diffraction characterisation

Powder X-ray diffraction data were collected on freshly prepared samples of $\mathbf{Mg_{0.5}}$, $\mathbf{Mg_1}$ and $\mathbf{Mg_2}$, on a Malvern Panalytical Empyrean multi-purpose platform diffractometer at the University of Glasgow, equipped with a Cu sealed tube X-ray source (λ (CuKα) = 1.5405 Å), at room temperature from 5 – 100° 2θ on zero-background silicon sample holders.

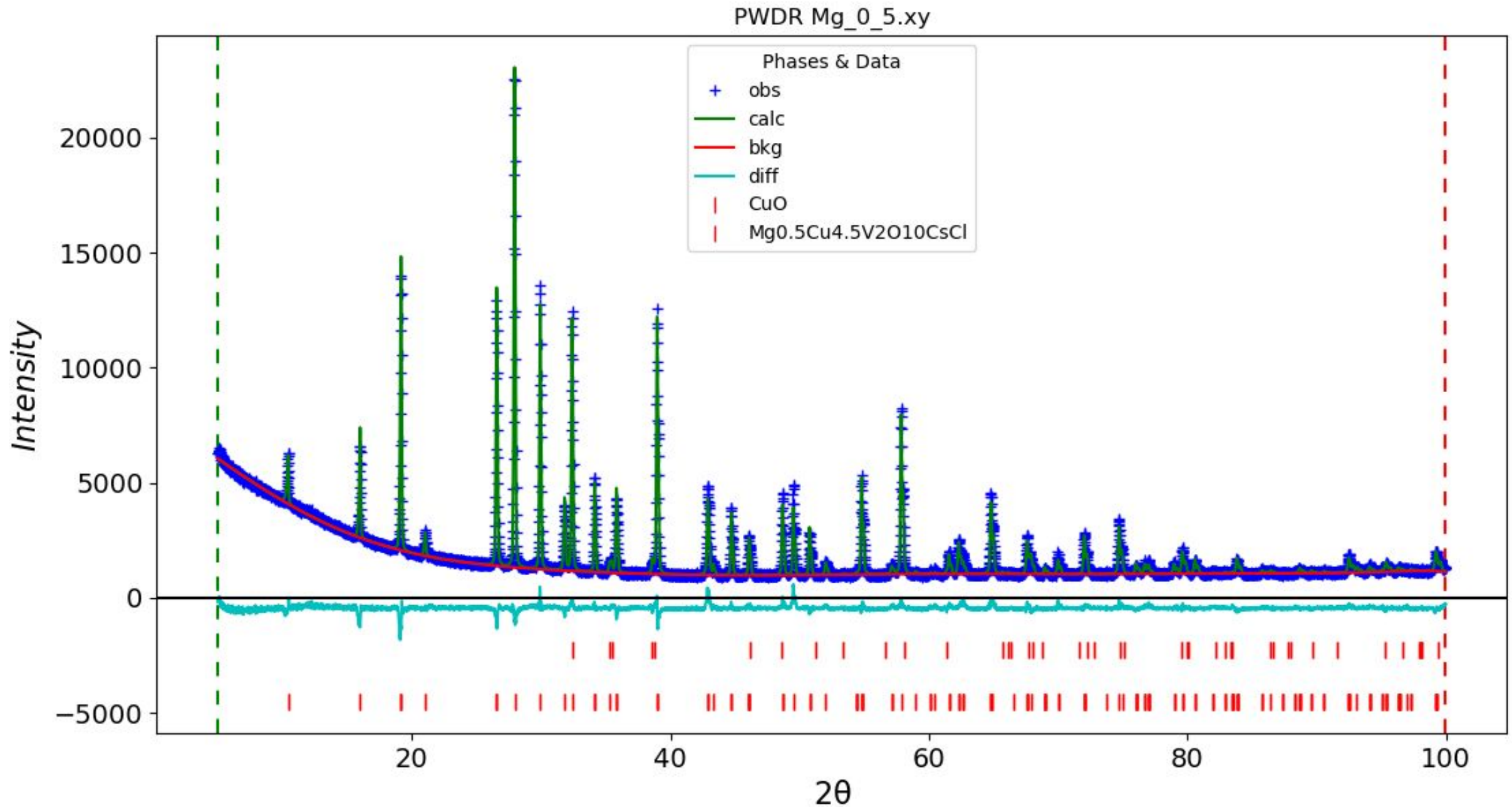

Figure S1 Experimental PXPD pattern of $\mathbf{Mg_{0.5}}$ *recorded on the Malvern Panalytical Empyrean multi-purpose platform diffractometer at room temperature. Shown are the calculated Rietveld fit to the data, difference plot and reflection positions, which are represented by the vertical tick marks. The goodness-of-fit is χ 2 = 3.929 and weighted R-value is Rw = 4.244.*

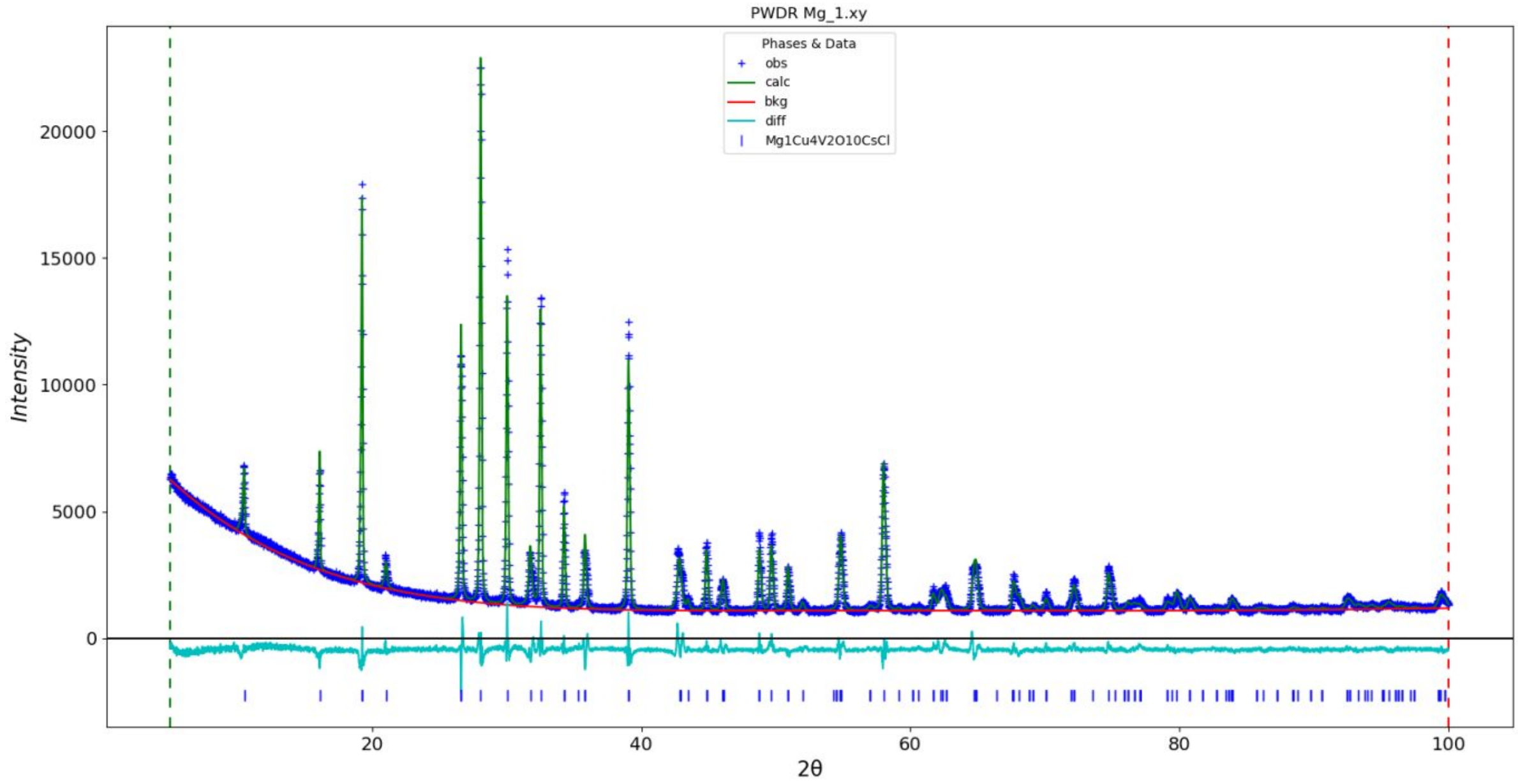

Figure S2 Experimental PXPD pattern of ***Mg_1*** *recorded on the Malvern Panalytical Empyrean multi-purpose platform diffractometer at room temperature. Shown are the calculated Rietveld fit to the data, difference plot and reflection positions, which are represented by the vertical tick marks. The goodness-of-fit is χ 2 = 5.711 and weighted R-value is Rw = 5.396.*

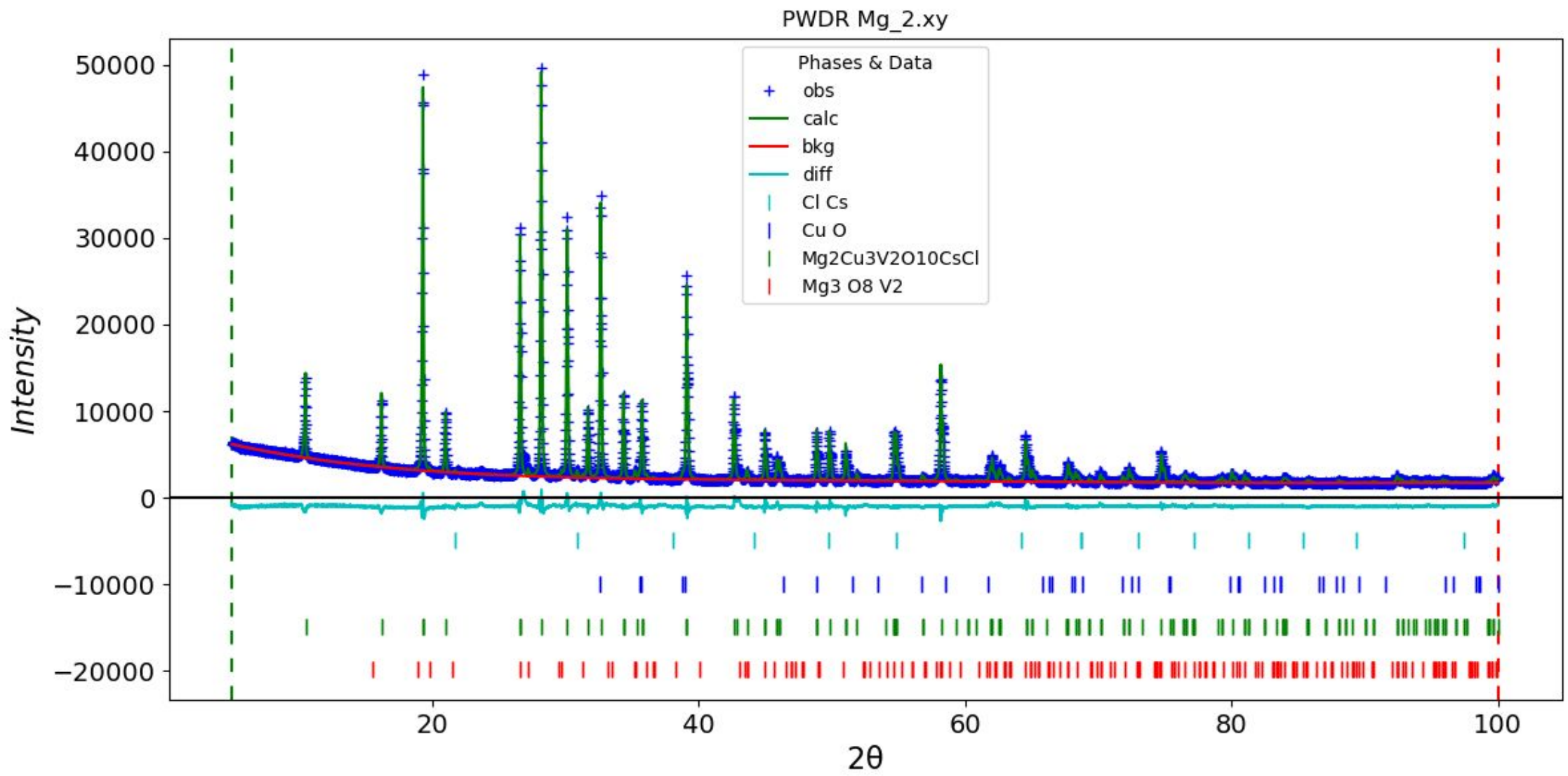

Figure S3 Experimental PXPD pattern of ***Mg_2*** *recorded on the Malvern Panalytical Empyrean multi-purpose platform diffractometer at room temperature. Shown are the calculated Rietveld fit to the data, difference plot and reflection positions, which are represented by the vertical tick marks. The goodness-of-fit is χ 2 = 7.220 and weighted R-value is Rw = 4.868.*

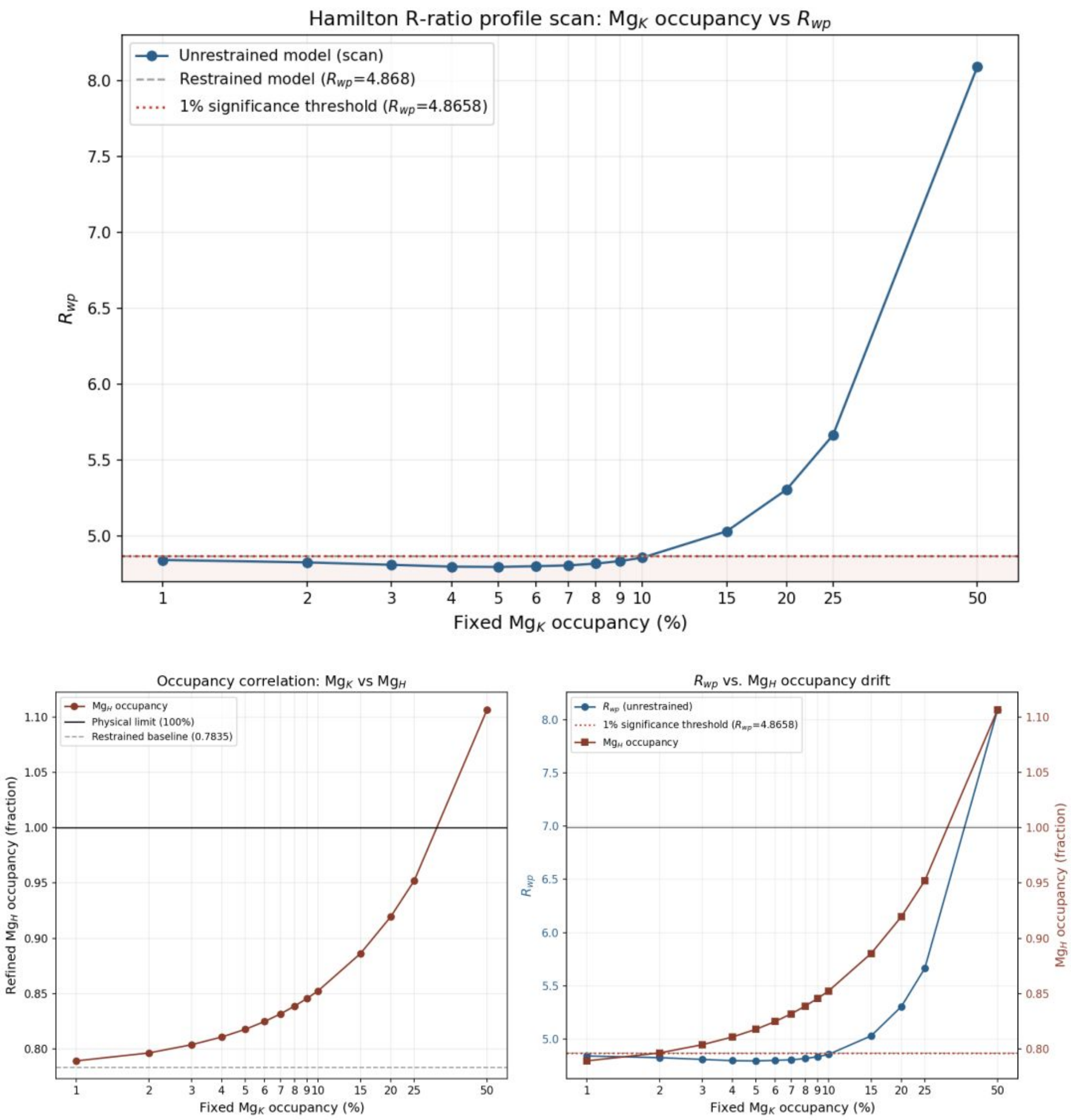

Figure S4 Top: Hamilton R-ratio profile scan of R_{wp} against fixed Mg_K occupancy, with the restrained-model R_{wp} and 1% significance threshold indicated. Bottom: Mg_H occupancy correlates strongly with fixed Mg_K occupancy (left), exceeding the physical limit of unity above MgK ≈ 30%; overlaying R_{wp} and Mg_H occupancy (right) shows the two compensating across the nominally significant region.

The Hamilton R-ratio test compares the goodness-of-fit of a restrained model (fewer parameters) against an unrestrained model (more parameters) via $R = R_{wp\,(restrained)}/R_{wp\,(unrestrained)}$, where $R_{wp}$is the weighted-profile R-factor. This ratio is judged significant at level αif it exceeds the critical value R_{crit}, where $R_{crit} = \sqrt{1 + F_{crit}(b,n-m,\alpha)/(n-m)}$, where n is the number of powder data points, m is the number of parameters in the unrestrained model, b is the number of additional parameters in the unrestrained model relative to the restrained model, and F_{crit} is the critical value of the F-distribution with $(b,n-m)$ degrees of freedom.

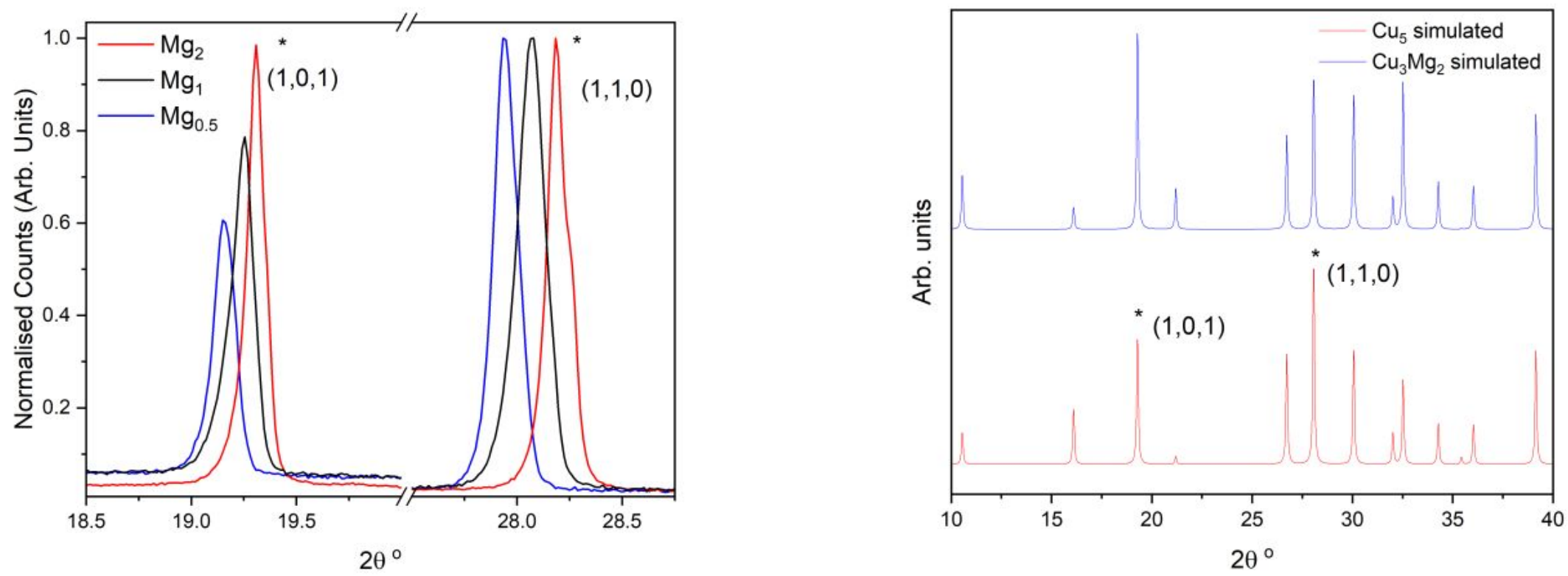

*Figure S5 A comparison of the (1,0,1) and (1,1,0) reflections obtained from the PXRD measurements of **$Mg_{0.5}$, Mg_1** and **Mg_2** , highlighting the decrease in intensity of the (1,1,0) plane with respect to the (1,0,1) plane (left) and a comparison of simulated XRD patterns from a single crystal of Cu_5-Averievite (red) and a structure where the two Cu_H sites have been substituted for Mg atoms (blue) (right).*

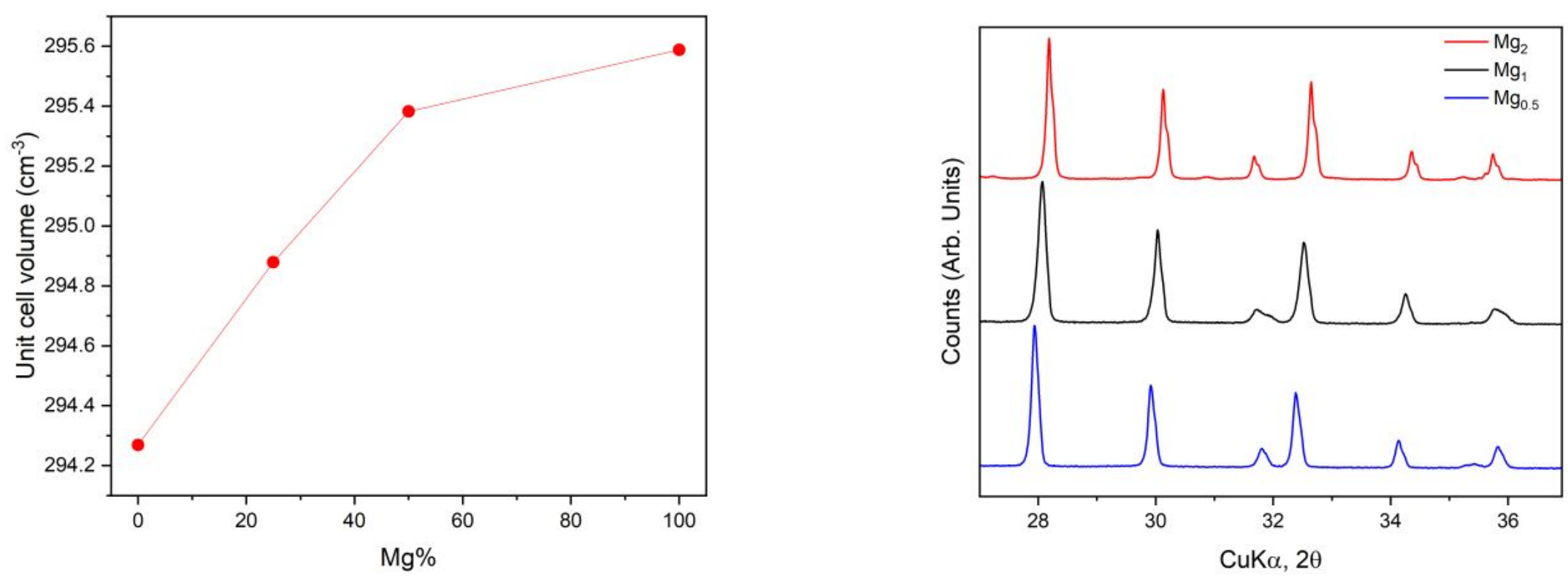

*Figure S6 (a) A comparison of the unit cell volume obtained from the PXRD refinements of **$Mg_{0.5}$, Mg_1** and **Mg_2** and (b) a comparison of the PXRD patterns o **$Mg_{0.5}$, Mg_1** and **Mg_2** between 27.5 – 36.5 2Θ, with a clear broadening of the 0,0,3 peak at ~31.8^o.*

Electron diffraction characterisation

The sample were prepared as a dark brown microcrystalline powder. For structural characterization with 3D ED, the dry powder was deposited on a 200-mesh copper TEM grid coated with a lacey film of ultrathin amorphous carbon by shaking the grid alongside powder and tapping off the excess.

3D-ED measurements were performed on a Rigaku XtaLAB Synergy-ED[S1] equipped with a LaB_6 source operating at 200 kV (λ= 0.0251 Å) at 10^{-5} Pa, and at a range of temperatures, between 100, 200, 250 and 300 K, using a Gatan Elsa Cryoholder.

Where data were collected at low temperature the samples were transferred to the cryoholder and into the diffractometer at room temperature before cooling. Diffraction patterns were collected for at least four crystals per sample at each temperature using a Rigaku HyPix-ED detector during continuous rotation of the crystals over ca. 120 °, with each image having a rotation width of 0.25 ° and an exposure time of a two seconds per degree, using a selected area aperture with an apparent diameter of 2 µm. The program CrysAlisPro[S2] was used to control the data collection and to process the diffraction data, for indexing, data reduction and space group determination. Crystallographic details are contained in the deposited CIFs for each of the structures reported and selected details are given below.

For each dataset, an initial structural model from previous XRD data was used as the start point for the least-squares refinement using kinematic approximation, performed with SHELXL[S3] or olex2.refine[S4] using scattering factors for electrons.[S5] An extinction parameter was refined to mitigate the effects of multiple diffraction. Anisotropic atomic displacement parameters (ADPs) were refined for all atoms with suitable restraints applied (rigid bond RIGU and similarity restraints SIMU in some cases). The Mg occupancy was refined competitively against the Cu_H site occupancy and the atomic positions and atomic displacement parameters (ADPs) constrained (EADP, EXYZ) to be exactly the same for the Cu and Mg atoms in question, except for **$Mg_{0.5}$** at 250K which was fixed at the value obtained from refinement against the data for the same sample at 298K. The resulting occupancies are reported in Tables S1-S4 below.

and CCDC 2559045-2559054 and 2558898 contain the supplementary crystallographic data for this paper. These data can be obtained free of charge from The Cambridge Crystallographic Data Centre via www.ccdc.cam.ac.uk/structures.

Upon cooling **Mg_0** below 295 K a symmetry breaking transition from the trigonal $P\bar{3}m1$ form to the monoclinic $P2_1/c$ space group is observed. Precession images for the hk0 plane (trigonal indexing and axes marked) are shown below in Figure S5 for data collected at 298K (left) and 250K (right). The additional peaks consistent with the monoclinic indexing are clearly visible.

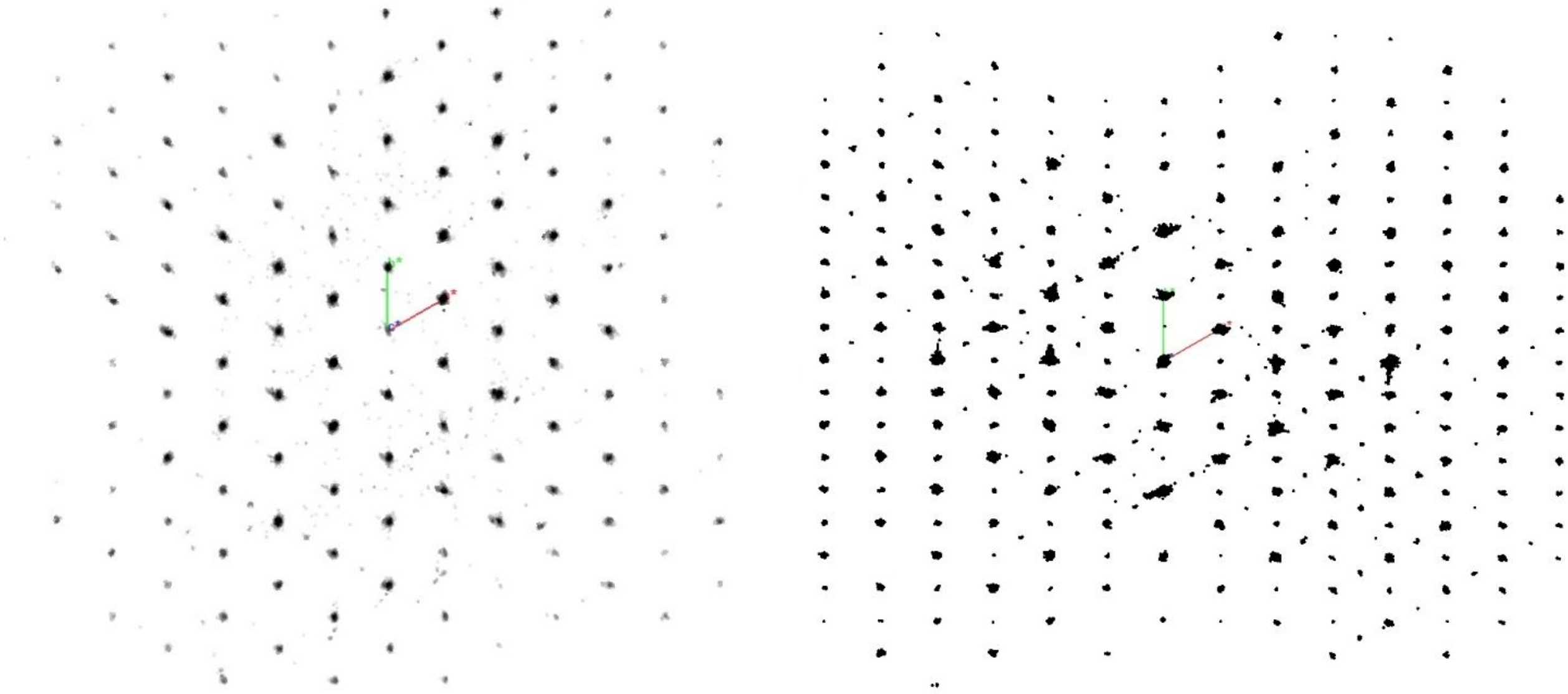

Figure S7 Precession images for the hk0 plane (trigonal indexing and axes marked) are shown for data collected at 298K (left) and 250K (right).

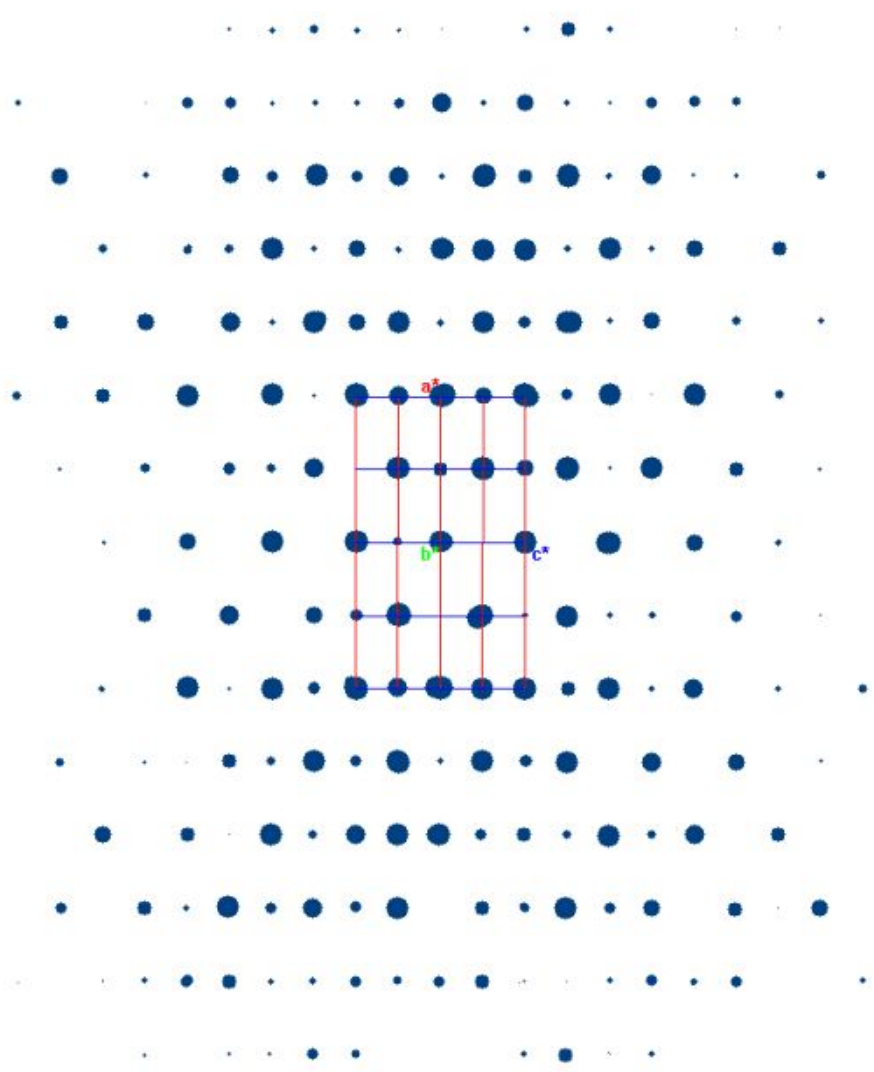

Figure S8 The monoclinic lattice overlaid over an equivalent view of the reconstructed reciprocal lattice, viewed down the 8.37Å unit cell direction (c axis for trigonal cell and monoclinic b axis for monoclinic form).

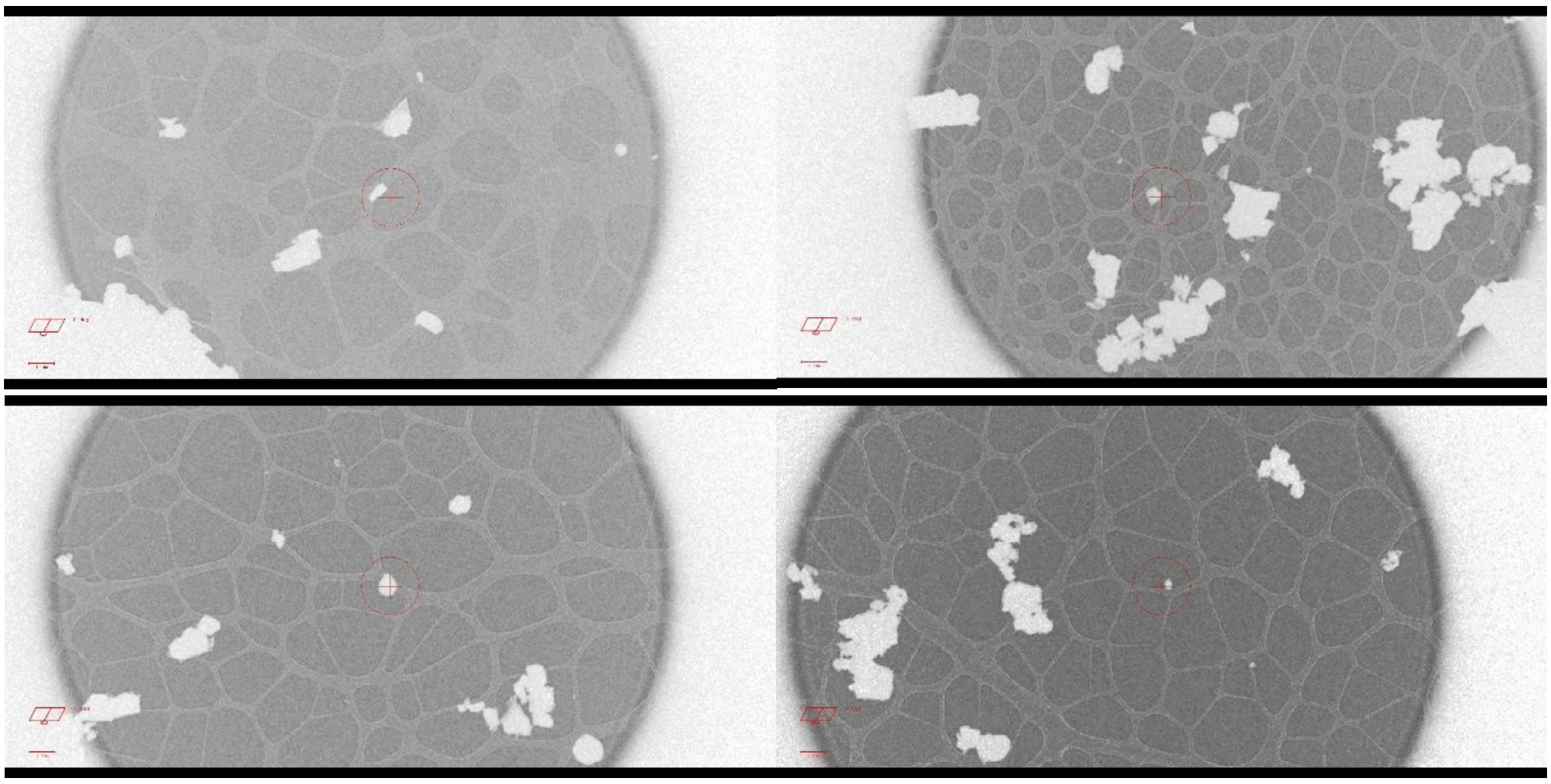

*Figure S9 Images of the crystals used for each electron diffraction experiment for each sample **Mg_0** (top left), **$Mg_{0.5}$** (top right), **Mg_1** (bottom left) and **Mg_2** (bottom right) at 300 K.*

*Table S1 Crystal Structure and refinement parameters obtained at 298 K from refinement of single crystal electron diffraction data 298 K for **Mg_1**.*

**298 K ED datasets**	**Mg_1**	**Mg_1**	**Mg_1**	**Mg_1**
System	Trigonal	Trigonal	Trigonal	Trigonal
Space group	$P\bar{3}m1$	$P\bar{3}m1$	$P\bar{3}m1$	$P\bar{3}m1$
a,b (Å)	6.3704(10)	6.3657(8)	6.3664(8)	6.3669(8)
c (Å)	8.4253(13)	8.4247(16)	8.4374(18)	8.4405(14)
α (°),β (°),γ (°)	90, 90, 120			
Volume (cm^{-3})	296.10(8)	295.65	296.16(8)	296.31(7)
Refined Mg %	29(4)	35(4)	0	17(4)
R_1%	12.29	12.23	13.60	11.66
wR_2%	28.67	29.66	32.52	27.68
Completeness %	84.9	93.7	79.3	92.5
Identifier	1797	1801	1800	1804
CCDC number	2559050	2559051	2559049	2559052

Table S2 Crystal Structure and refinement parameters obtained at 298 and 250 K from refinement of single crystal electron diffraction data for ***Mg_0***.

**Mg_0**	298 K	250 K
System	Trigonal	Monoclinic
Space group	$P\bar{3}m1$	$P2_1/c$
a (Å)	6.3775 (16)	8.3780(13)
b (Å)	6.3775(16)	6.3805(9)
c (Å)	8.3769(13)	11.048(5)
α (°)	90	90
β (°)	90	90.11(2)
γ (°)	120	90
Volume (Å^3)	295.07(13)	590.6(3)
Refined Mg %	0	0
R_1%	12.04	14.27
wR_2%	30.93	38.30
Completeness%	97.4	73.7
Identifier	2191	2204
CCDC number	2559045	2559046

Table S3 Crystal Structure and refinement parameters obtained at 298 and 250 K from refinement of single crystal electron diffraction data for ***$Mg_{0.5}$***. ** The Mg occupancy was fixed to the value obtained for the 298 K dataset.*

**$Mg_{0.5}$**	298 K	250 K
System	Trigonal	Trigonal
Space group	$P\bar{3}m1$	$P\bar{3}m1$
a,b (Å)	6.3744(10)	6.3661(8)
c (Å)	8.3977(12)	8.4275(19)
α (°),β (°),γ (°)	90, 90, 120	
Volume (Å^3)	295.59(8)	295.78(9)
Refined Mg %	17(4)	17*
R_1 %	13.28	13.54
wR_2%	30.71	31.95
Completeness%	93.7	84.1
Identifier	1806	1817
CCDC number	2559047	2559048

Table S4 Crystal Structure and refinement parameters obtained at 298, 250 and 200 K from refinement of single crystal electron diffraction data for ***Mg_2****.*

**Mg_2**	298 K	200 K	100 K
System	Trigonal	Trigonal	Trigonal
Space group	$P\bar{3}m1$	$P\bar{3}m1$	$P\bar{3}m1$
a,b (Å)	6.3618(9)	6.3663(10)	6.3536(9)
c (Å)	8.4946 (13)	8.4840(14)	8.495(2)
α (°),β (°),γ (°)	90, 90, 120		
β (°)	90	90	90
γ (°)	120	120	120
Volume (Å^3)	297.74 (8)	297.79(8)	297.0(1)
Refined Mg %	84(3)	87(4)	84(4)
R_1 %	12.92	12.23	12.71
wR_2 %	30.01	28.99	31.03
Completeness	95.4	95.9	88.8
Identifier	1781	1983	1981
CCDC number	2559053	2558898	2559054

Table S6 Full crystallographic tables for ***Mg_0*** *and* ***$Mg_{0.5}$****collected at variable temperatures.*

**Compound**	**Mg_0**	**Mg_0**	**$Mg_{0.5}$**	**$Mg_{0.5}$**

Formula	$ClCsCu_5O_{10}V_2$	$ClCsCu_5O_{10}V_2$	$ClCsCu_{4.66}Mg_{0.34}O_{10}V_2$	$ClCsCu_{4.66}Mg_{0.34}O_{10}V_2$
$D_{calc.}$/ g cm^{-3}	4.209	4.206	4.128	4.124
Formula Weight	747.967	747.9645	734.625	734.625
Colour	brown	brown	brown	brown
Shape	plate	plate	plate	plate
Size/mm^3	0.0007 × 0.0002 × 0.0002	0.0002×0.0002×0.0001	0.0005×0.0004×0.0002	0.0005×0.0004×0.0002
T/K	298.00(10)	250.00(10)	298.00(10)	244.00(10)
Crystal System	trigonal	monoclinic	trigonal	trigonal
Space Group	*P*-3*m*1	$P2_1/c$	*P*-3*m*1	*P*-3*m*1
a/Å	6.3775(16)	8.3780(13)	6.3744(10)	6.3661(8)
b/Å	6.3775(16)	6.3805(9)	6.3744(10)	6.3661(8)
c/Å	8.3769(19)	11.048(5)	8.3977(12)	8.4275(19)
a/°	90	90	90	90
b/°	90	90.11(2)	90	90
g/°	120	90	120	120
V/Å^3	295.07(13)	590.6(3)	295.50(8)	295.78(9)
Z	1	2	1	1
Z'	0.083333	0.5	0.083333	0.083333
Wavelength/Å	0.02510			
Radiation type	electron			
θ_{min}/°	0.13	0.09	0.13	0.13
θ_{max}/°	0.85	0.90	0.90	0.90
Index ranges	$-7 \le h \le 7$, $-7 \le k \le 7$, $-10 \le l \le 10$	$-10 \le h \le 10$, $-7 \le k \le 7$, $-10 \le l \le 10$	$-7 \le h \le 7$, $-7 \le k \le 7$, $-10 \le l \le 10$	$-7 \le h \le 7$, $-7 \le k \le 7$, $-9 \le l \le 9$
Measured Refl.	1381	2532	1062	1436
Independent Refl.	220	890	259	219
Reflections with I≥s(I)	198	587	253	203
R_{int}	0.2003	0.1308	0.0760	0.1505
Parameters	29	101	30	29
Restraints	0	6	0	0
Largest Peak	0.8467	0.8737	0.5697	0.4743
Deepest Hole	-0.6378	-1.6772	-0.7915	-0.5242
GooF	1.0883	1.2544	1.0586	1.1518
wR_2 (all data)	0.3091	0.3830	0.3467	0.3194
wR_2	0.2980	0.3605	0.3454	0.3153
R_1 (all data)	0.1318	0.1761	0.1468	0.1381
R_1	0.1204	0.1427	0.1448	0.1354
CCDC number	2559045	2559046	2559047	2559048

Table S7 Full crystallographic tables for four different $\mathbf{Mg_1}$ *crystallites collected at 298 K.*

**Compound**	$\mathbf{Mg_1}$	$\mathbf{Mg_1}$	$\mathbf{Mg_1}$	$\mathbf{Mg_1}$

Formula	$ClCsCu_{4.3}Mg_{0.69}O_{10}V_2$	$ClCsCu_{4.42}Mg_{0.58}O_{10}V_2$	$ClCsCu_5O_{10}V_2$	$ClCsCu_{4.84}Mg_{0.16}O_{10}V_2$
$D_{calc.}$/ g cm^{-3}	4.045	4.067	4.194	4.156
Formula Weight	720.255	725.207	747.967	741.688
Colour	brown	brown	brown	brown
Shape	plate	plate	plate	plate
Size/mm^3	0.0008×0.007×0.0003	0.001×0.0002×0.0002	0.0012×0.001×0.0002	0.001×0.0004×0.0002
T/K	298.00(10)	298.00(10)	298.00(10)	298.00(10)
Crystal System	trigonal	trigonal	trigonal	trigonal
Space Group	*P*-3*m*1	*P*-3*m*1	*P*-3*m*1	*P*-3*m*1
a/Å	6.3657(8)	6.3704(10)	6.3664(8)	6.3669(8)
b/Å	6.3657(8)	6.3704(10)	6.3664(8)	6.3669(8)
c/Å	8.4247(16)	8.4253(13)	8.4374(18)	8.4405(14)
a/°	90	90	90	90
b/°	90	90	90	90
g/°	120	120	120	120
V/Å^3	295.65(8)	296.10(8)	296.16(8)	296.31(7)
Z	1	1	1	1
Z'	0.083333	0.083333	0.083333	0.083333
Wavelength/Å	0.02510			
Radiation type	electron			
θ_{min}/°	0.13	0.13	0.13	0.13
θ_{max}/°	0.90	0.90	0.90	0.90
Index ranges	$-7 \le h \le 7$, $-7 \le k \le 7$, $-10 \le l \le 10$	$-7 \le h \le 7$, $-6 \le k \le 6$, $-10 \le l \le 10$	$-7 \le h \le 7$, $-7 \le k \le 7$, $-9 \le l \le 9$	$-7 \le h \le 7$, $-7 \le k \le 7$, $-10 \le l \le 10$
Measured Refl.	1629	683	1447	1638
Independent Refl.	249	225	210	247
Reflections with I≥s(I)	238	218	202	240
R_{int}	0.1268	0.0719	0.1487	0.1137
Parameters	30	30	27	30
Restraints	0	0	3	0
Largest Peak	0.6710	0.9411	0.6108	0.6329
Deepest Hole	-0.6804	-0.8030	-0.9204	-0.6345
GooF	1.0484	1.0614	1.0650	1.0635
wR_2 (all data)	0.2962	0.2862	0.3252	0.2762
wR_2	0.2929	0.2844	0.3232	0.2744
R_1 (all data)	0.1263	0.1261	0.1380	0.1182
R_1	0.1224	0.1230	0.1360	0.1166
CCDC number	2559051	2559050	2559049	2559052

Table S8 Full crystallographic tables for ***Mg_2*** *collected at variable temperatures.*

**Compound**	**Mg_2**	**Mg_2**	**Mg_2**
Formula	$ClCsCu_{3.31}Mg_{1.68}O_{10}V_2$	$ClCsCu_{3.26}Mg_{1.74}O_{10}V_2$	$ClCsCu_{3.32}Mg_{1.68}O_{10}V_2$
$D_{calc.}$/ g cm^{-3}	3.800	3.790	3.813

Formula Weight	681.407	679.688	682.042
Colour	brown	brown	brown
Shape	plate	plate	plate
Size/mm³	0.0003×0.0002×0.0002	0.0002×0.0002×0.0002	0.0007×0.0006×0.0002
T/K	298.00(10)	200.60(10)	101.00(10)
Crystal System	trigonal	trigonal	trigonal
Space Group	*P*-3*m*1	*P*-3*m*1	*P*-3*m*1
a/Å	6.3618(9)	6.3663(10)	6.3536(9)
b/Å	6.3618(9)	6.3663(10)	6.3536(9)
c/Å	8.4946(13)	8.4840(14)	8.495(2)
a/°	90	90	90
b/°	90	90	90
g/°	120	120	120
V/Å³	297.74(8)	297.79(8)	297.0(1)
Z	1	1	1
Z'	0.083333	0.083333	0.083333
Wavelength/Å	0.02510		
Radiation type	electron		
$\theta_{min}/°$	0.08	0.13	0.13
$\theta_{max}/°$	0.90	0.90	0.90
Index ranges	-7 ≤ *h* ≤ 7, -7 ≤ *k* ≤ 7, -10 ≤ *l* ≤ 10	-7 ≤ *h* ≤ 7, -7 ≤ *k* ≤ 7, -10 ≤ *l* ≤ 10	-7 ≤ *h* ≤ 7, -7 ≤ *k* ≤ 7, -9 ≤ *l* ≤ 9
Measured Refl.	1526	1125	1463
Independent Refl.	253	253	233
Reflections with I≥s(I)	244	237	222
R_{int}	0.1655	0.1292	0.1431
Parameters	30	30	30
Restraints	0	0	0
Largest Peak	0.6528	0.7603	0.6665
Deepest Hole	-0.7724	-1.2203	-0.6495
GooF	1.0762	1.0721	1.0569
wR_2 (all data)	0.3001	0.2890	0.3102
wR_2	0.2961	0.2838	0.3056
R_1 (all data)	0.1335	0.1288	0.1331
R_1	0.1292	0.1228	0.1276
CCDC number	2559053	2558898	2559054

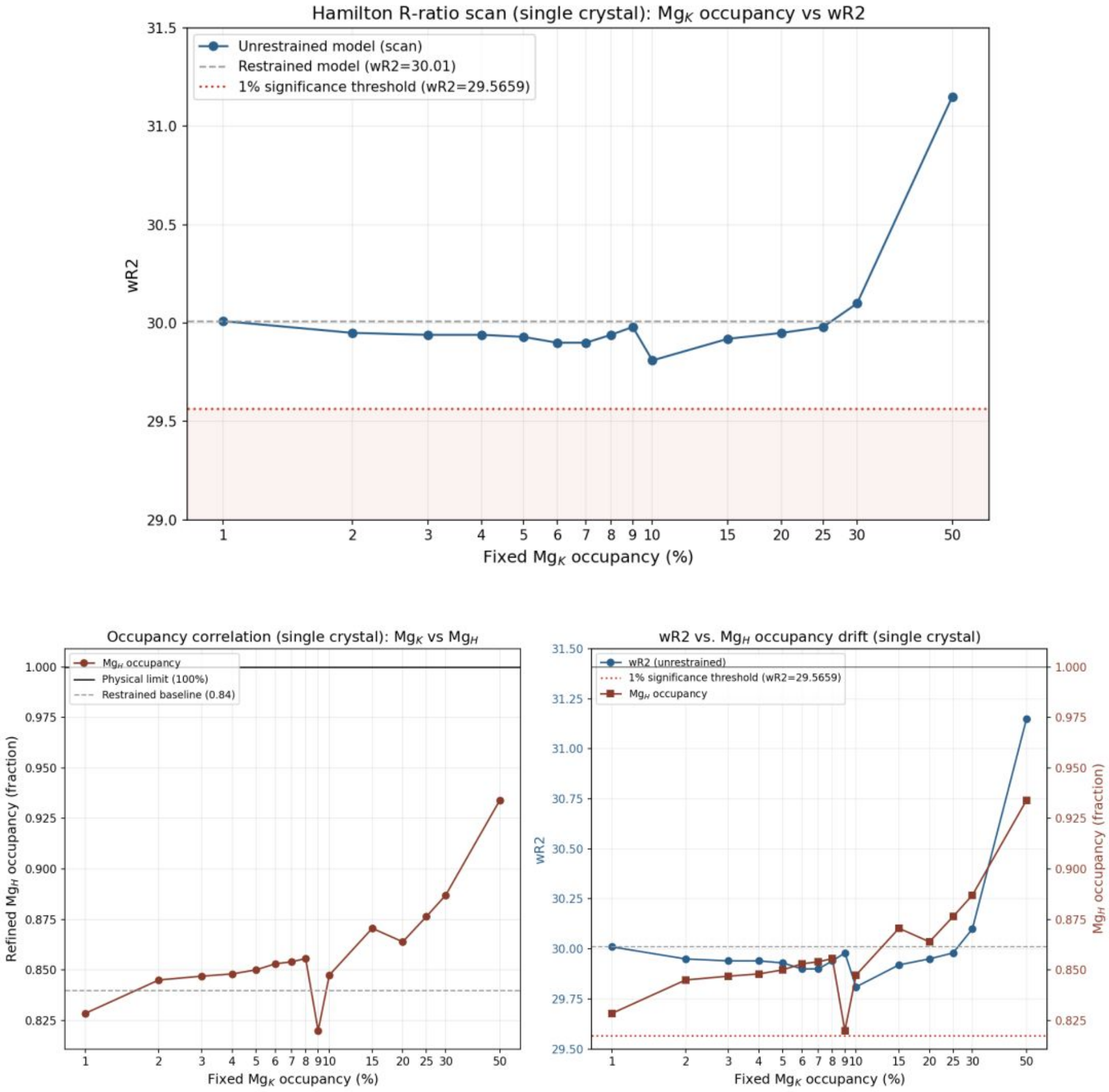

Figure S10 Equivalent Hamilton R-ratio scan applied to the single-crystal refinement (wR2 against fixed Mg_K occupancy); no point exceeds the 1% significance threshold (top). A plot of Mg_H occupancy vs fixed Mg_K occupancy (left), and overlaying wR2 with MgH occupancy (right) shows the same compensating trend seen in the powder data, despite the absence of formal statistical significance in this dataset.

SEM-EDX characterisation

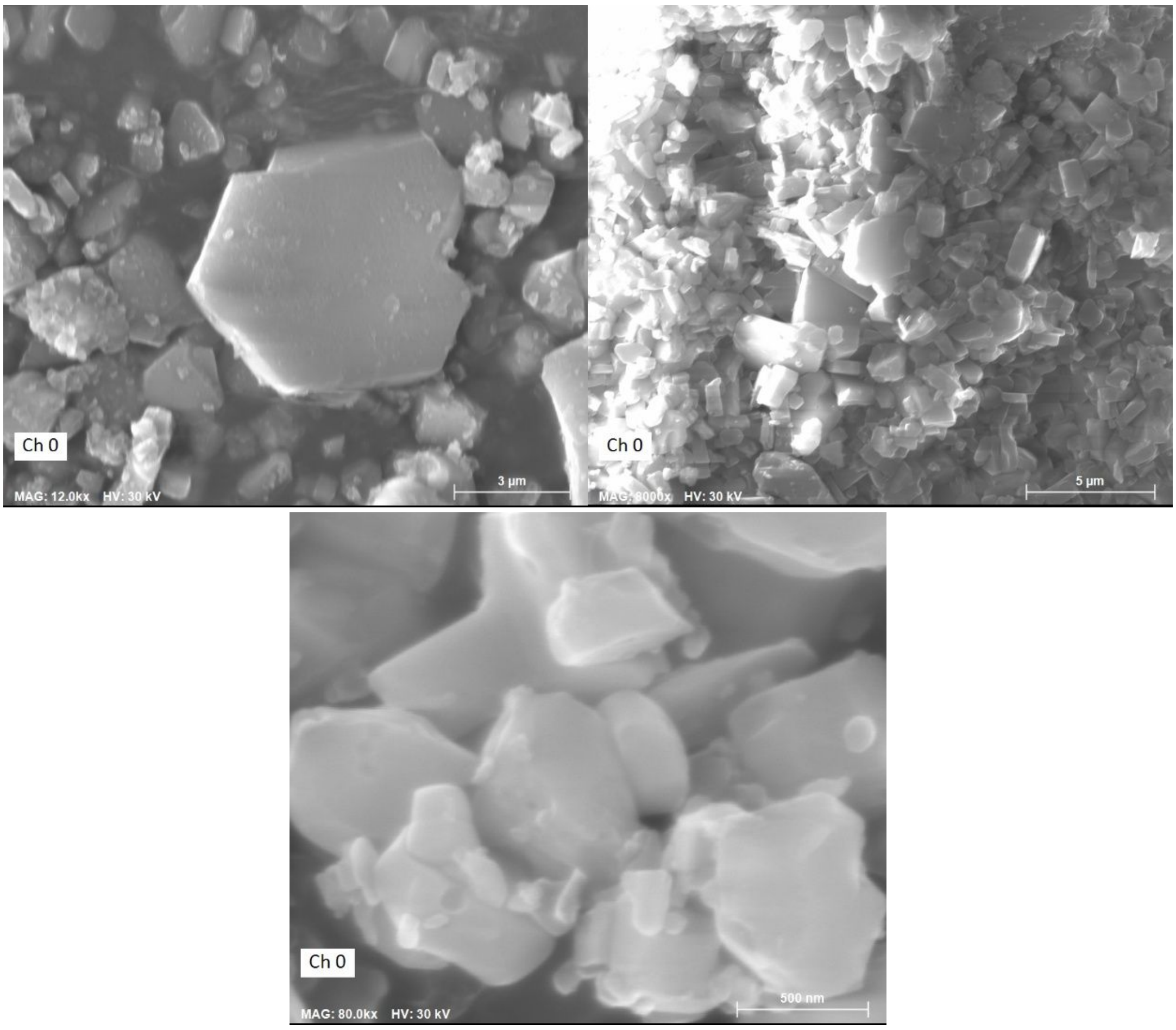

*Figure S11 SEM images obtained at 30 kV and 12,000, 8,000 and 80000 x magnification for **$Mg_{0.5}$** (top left), **Mg_1** (top right) and **Mg_2** (bottom), respectively.*

*Table S9 A comparison of the expected and calculated atomic percentages for samples **$Mg_{0.5}$, Mg_1, Mg_2** obatined form SEM-EDX.*

	Expected (Mg_0)	Expected (Mg_2)	**$Mg_{0.5}$** Atomic %	**Mg_1** Atomic %	**Mg_2** Atomic %
Copper	26.32	15.78	21.85(55)	20.37(52)	17.74(46)
Magnesium	0	10.53	2.64(20)	4.65(32)	9.67(63)
Vanadium	10.53	10.53	12.30(35)	12.30(35)	12.34(36)
Cesium	5.26	5.26	5.34(15)	5.79(17)	5.93(18)
Chlorine	5.26	5.26	5.08(21)	5.76(23)	4.53(21)
Oxygen (fixed)	52.63	52.63	52.80	51.14	49.94

Magnetic characterisation

Variable-temperature, solid-state dc and ac magnetic susceptibility data were collected on a Quantum Design MPMS3 SQUID magnetometer at the University of Glasgow. Microcrystalline

samples were prepared using a mortar and pestle in open air and then added to gelatin capsules within a straw. Diamagnetic corrections for the straw and geletin capsule were determined by blank measurements. Additional diamagnetic corrections were applied to the observed paramagnetic susceptibilities using Pascal's constants.

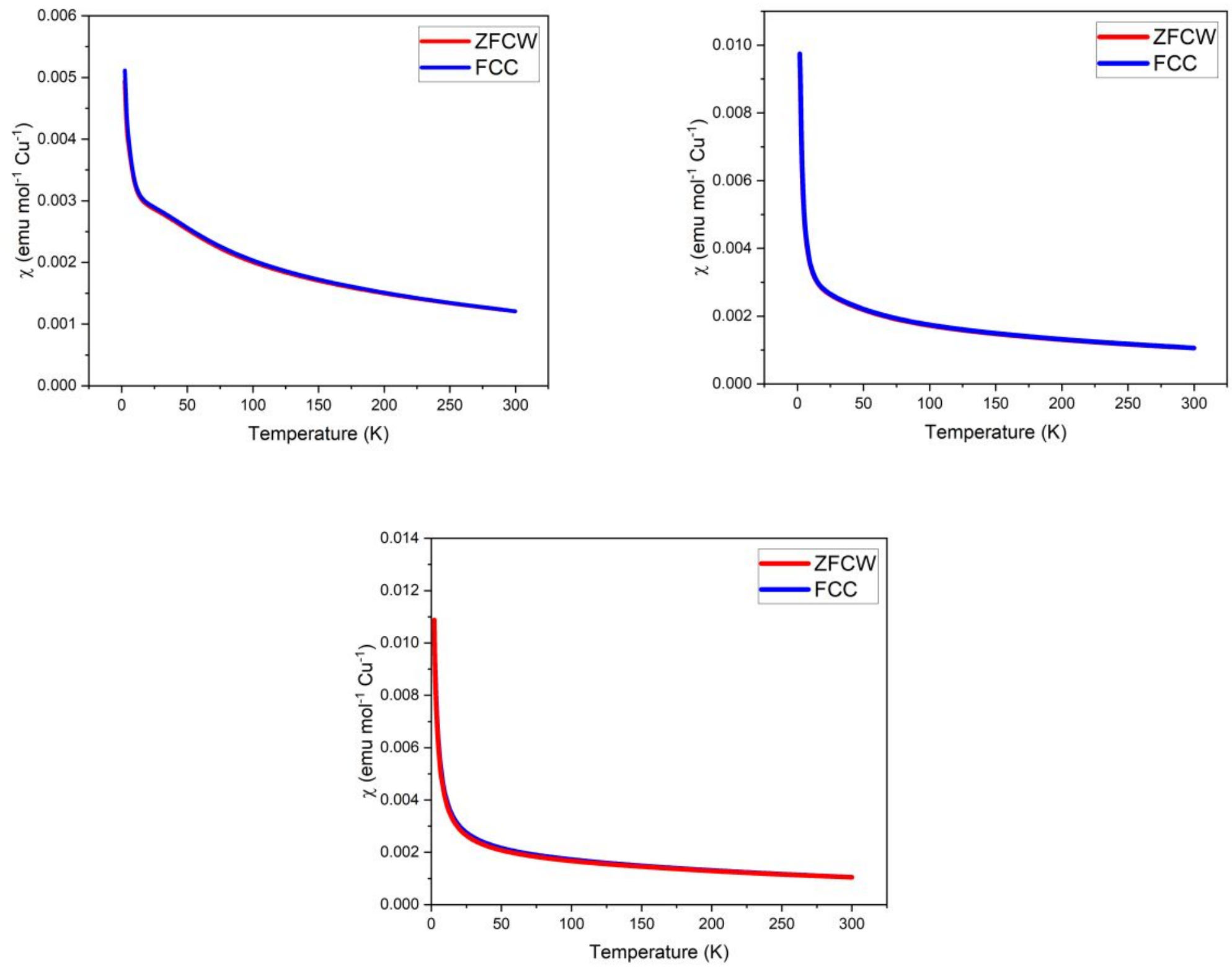

*Figure S12 Field-cooled and zero field-cooled magnetic susceptibility for compounds **$Mg_{0.5}$** (left), **Mg_1** (right) and **Mg_2** (bottom).*

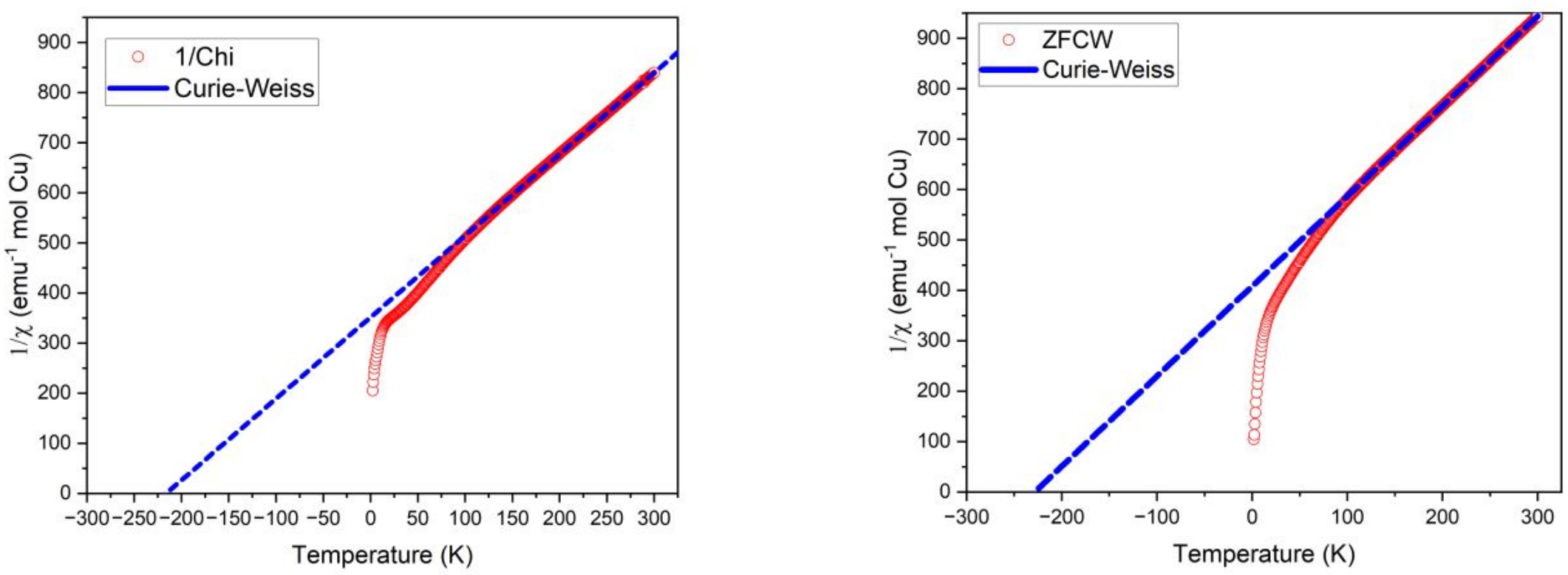

*Figure S13 Inverse magnetic susceptibility for compounds **$Mg_{0.5}$** (left) and **Mg_1** (right) and the corresponding Curie-Weiss fits (blue dashed lines).*

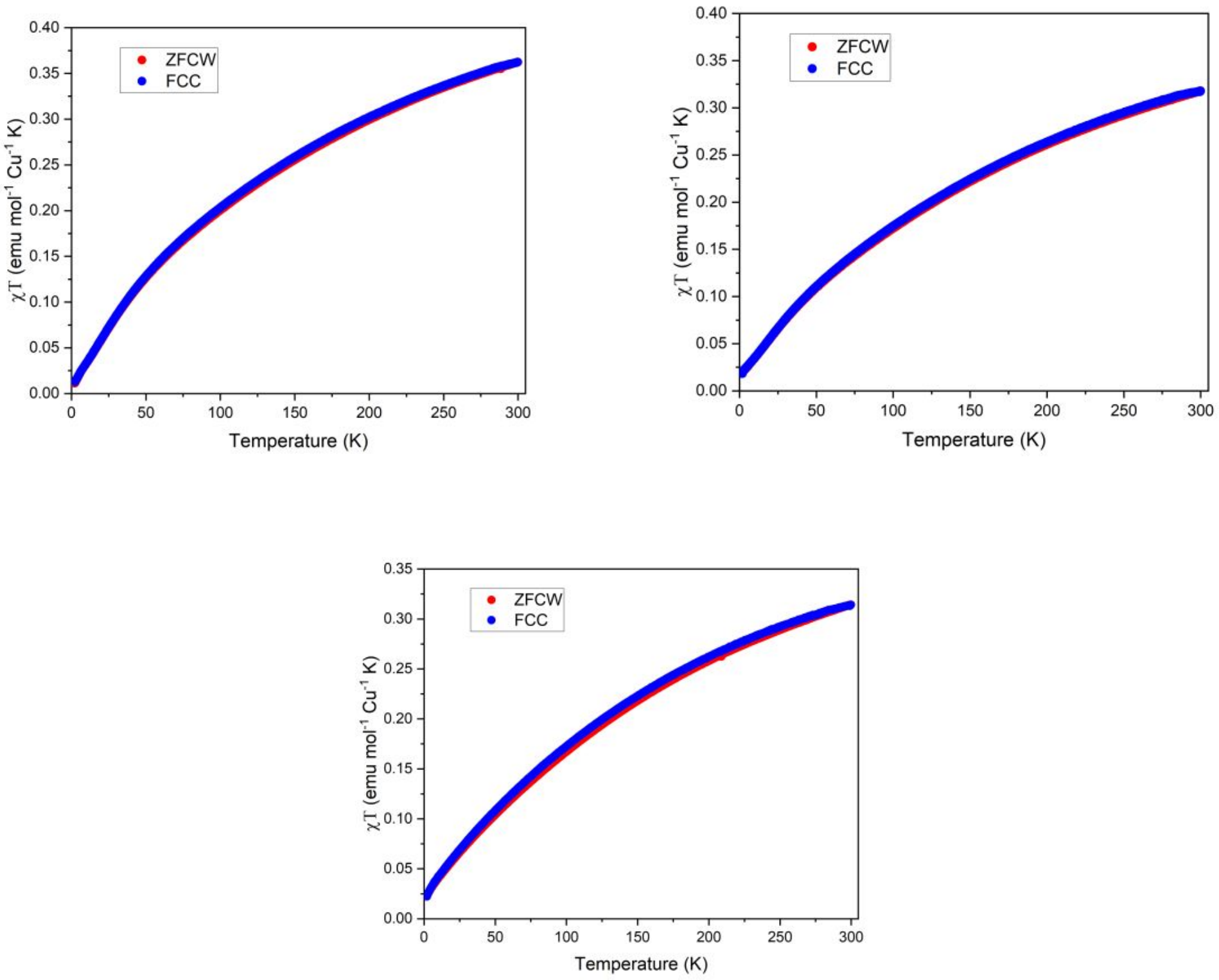

*Figure S14 X_T vs T plots for compounds **$Mg_{0.5}$** (left), **Mg_1** (right) and **Mg_2** (bottom) from 2 – 300 K.*

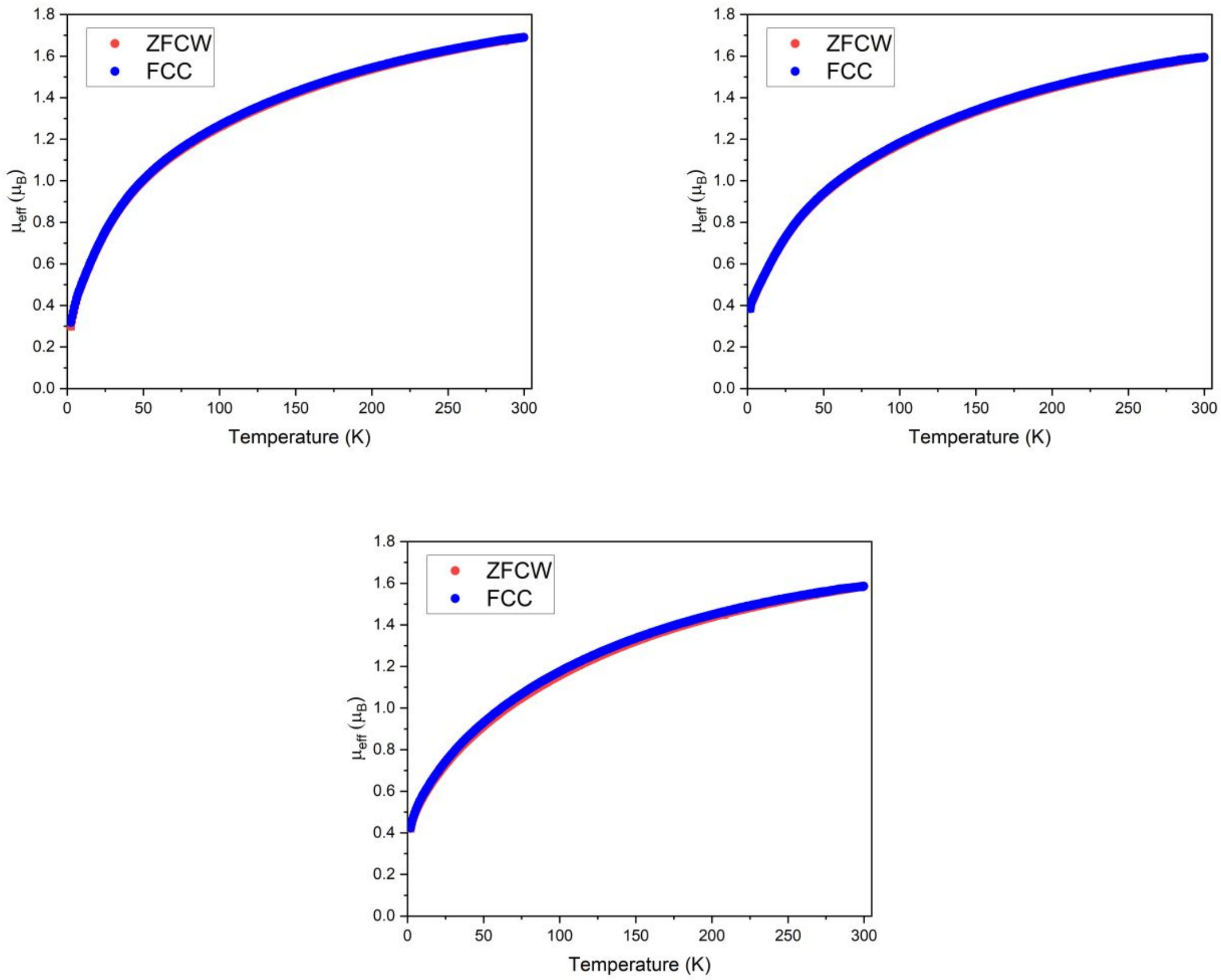

*Figure S15 μ_{eff} vs T plots for compounds **$Mg_{0.5}$** (left), **Mg_1** (right) and **Mg_2** (bottom) from 2 – 300 K*

Magnetization data from **Mg_2** and **Zn_2** at T = 2 K as a function of field are shown in Figure 4, where the intrinsic kagome contribution and a paramagnetic-like response from defect Cu spins can be disentangled using equation 1.[S6]

$$M = nM_{sat}\tanh(gS\mu_B H/ k_B(T+\theta)) + \chi_{kag}H \quad (1)$$

Where S = 1/2 and M_{sat} is the saturated magnetization of 1 mole of Cu^{2+} spins given by $N_A g \mu_B S$. The first term of the equation accounts for the defect spin magnetization, using an adapted Brillouin function that describes paramagnetic-like spins with a weak coupling given by the energy scale of θ. The concentration of defect spins in the sample is given by n. The second term of equation 1 accounts for the intrinsic magnetization of the kagome lattice. It is assumed that the kagome exchange interactions are much larger than the applied field and that the intrinsic kagome susceptibility is constant with increasing field within the measurement range. Figure S13 shows the magnetization per Cu^{2+} ion, normalized to the saturated magnetization of 1 mole of S = 1/2 spins and fitted with equation 1. The g-factor was fixed to g = 2.45 derived from μeff = 2.12 μ_B. The refined values of n and θ are 1.83(5)% and – 0.13(5) K, respectively, indicating less than 2 % of defect Cu^{2+} spins. Comparing these parameters to those obtained for the **Zn_2** analogue shows a significant decrease in the estimated number of defect spins from ~3%.[S6] The high level of similarity in the intrinsic contributions from the Kagome plane is further indication of the high level of doping in the **Mg_2** sample, resulting in two compounds which possess extremely similar well isolated Kagome planes.

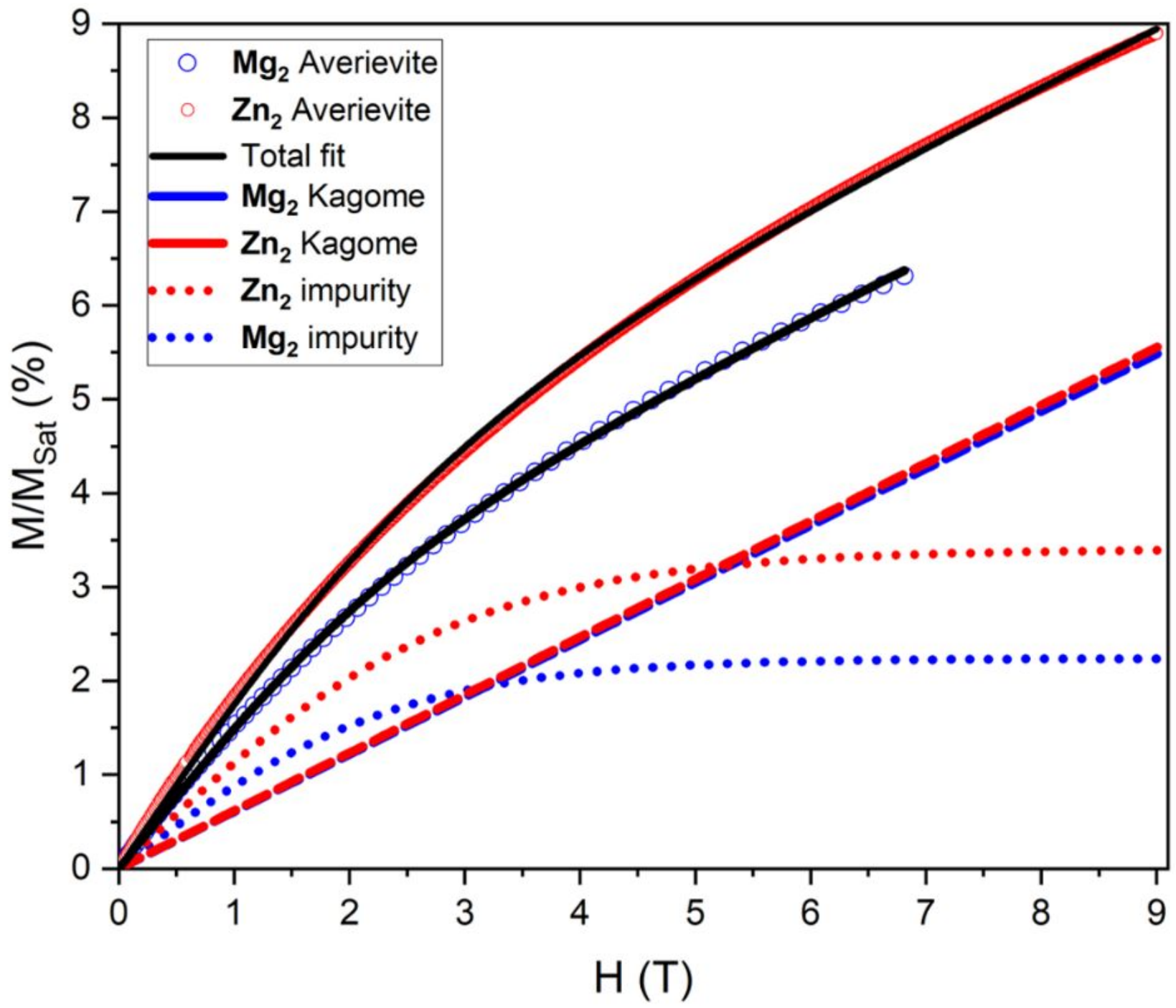

*Figure S16 Magnetisation vs field plots for **Mg_2** and **Zn_2** measured at T = 2 K, normalized to 1 mole of Cu2+ spins. The black lines are fits using equation 1, the red and blue dashed lines are the intrinsic kagome magnetisation and the red and blue dotted lines are the magnetization of defect Cu^{2+} spins.[S6]*

References

[S1] Ito, S.; White, F. J.; Okunishi, E.; Aoyama, Y.; Yamano, A.; Sato, H.; Ferrara, J. D.; Jasnowski, M.; Meyer, M. Structure Determination of Small Molecule Compounds by an Electron Diffractometer for 3D ED/MicroED. *CrystEngComm* **2021**, *23*, 8622–8630.

[S2] Rigaku Oxford Diffraction. *CrysAlisPro*, version 171.44.81a; Rigaku Corporation: Wroclaw, Poland, 2024.

[S3] Sheldrick, G. M. Crystal Structure Refinement with *SHELXL*. *Acta Crystallogr., Sect. C: Struct. Chem.* **2015**, *71*, 3–8.

[S4] Bourhis, L. J.; Dolomanov, O. V.; Gildea, R. J.; Howard, J. A. K.; Puschmann, H. The Anatomy of a Comprehensive Constrained, Restrained Refinement Program for the Modern Computing Environment — *Olex2* Dissected. *Acta Crystallogr., Sect. A: Found. Adv.* **2015**, *71*, 59–75.

[S5] Peng, L.-M. Electron Atomic Scattering Factors and Scattering Potentials of Crystals. *Micron* **1999**, *30*, 625–648.

[S6] Georgopoulou, M.; Boldrin, D.; Fåk, B.; Manuel, P.; Gibbs, A.; Ollivier, J.; Suard, E.; and Wills, A. S. Magnetic ground states and excitations in Zn-doped averieite – A

family of oxide-based S = 1/2 kagome antiferromagnets. *arXiv* **2023**, arXiv:2306.14739. DOI: 10.48550/arXiv.2306.14739.
